# Material‐Assisted Strategies for Osteochondral Defect Repair

**DOI:** 10.1002/advs.202200050

**Published:** 2022-03-24

**Authors:** Constance Lesage, Marianne Lafont, Pierre Guihard, Pierre Weiss, Jérôme Guicheux, Vianney Delplace

**Affiliations:** ^1^ Université de Nantes Oniris CHU Nantes INSERM Regenerative Medicine and Skeleton RMeS UMR 1229 Nantes F‐44000 France; ^2^ HTL Biotechnology 7 Rue Alfred Kastler Javené 35133 France

**Keywords:** biologics, biomaterials, cartilage, cell therapy, osteochondral defect, regenerative medicine, subchondral bone

## Abstract

The osteochondral (OC) unit plays a pivotal role in joint lubrication and in the transmission of constraints to bones during movement. The OC unit does not spontaneously heal; therefore, OC defects are considered to be one of the major risk factors for developing long‐term degenerative joint diseases such as osteoarthritis. Yet, there is currently no curative treatment for OC defects, and OC regeneration remains an unmet medical challenge. In this context, a plethora of tissue engineering strategies have been envisioned over the last two decades, such as combining cells, biological molecules, and/or biomaterials, yet with little evidence of successful clinical transfer to date. This striking observation must be put into perspective with the difficulty in comparing studies to identify overall key elements for success. This systematic review aims to provide a deeper insight into the field of material‐assisted strategies for OC regeneration, with particular considerations for the therapeutic potential of the different approaches (with or without cells or biological molecules), and current OC regeneration evaluation methods. After a brief description of the biological complexity of the OC unit, the recent literature is thoroughly analyzed, and the major pitfalls, emerging key elements, and new paths to success are identified and discussed.

## Graphical abstract

Bioengineering osteochondral tissues for osteochondral defect repair is a major biomedical challenge. While a broad variety of tissue engineering approaches have been proposed, little progress has been made. By scrutinizing the methodologies and outcomes of more than five years of research worldwide, a complete overview of the field is drawn, allowing to identify limitations and potential key elements for success.

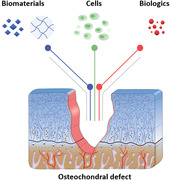

## 1 Introduction

Articular cartilage is a connective tissue present at the extremities of bones in diarthrodial joints, which has major functions in joint lubrication and stress reduction during movement.^[^ ^1^ ^]^ These functions are mediated by the highly hydrated and organized matrix of the tissue, whose integrity, defined according to the International Cartilage Repair Society (ICRS) classification, is essential for the maintenance of its biomechanical and diffusion properties.^[^ ^2^ ^]^ These properties can be altered if the complex structure of cartilage is damaged by repetitive excessive loading, trauma, or diseases, leading to cartilage defects. These defects can vary in a range from grade I (nearly normal) to grade IV (severely abnormal), depending on the depth of the defect and whether it affects cartilage only or cartilage and subchondral bone, that is, the whole osteochondral (OC) unit.^[^ ^3^ ^]^ Several retrospective studies revealed the presence of defects of all grades (grades II and III being the most common) in more than 60% of patients of all ages who underwent an arthroscopy.^[^ ^4^, ^5^ ^]^ As these defects are often asymptomatic,^[^ ^6^ ^]^ they are difficult to diagnose and treat before further degeneration, which can culminate in the onset of osteoarthritis.

The first surgical approaches for the management of cartilage defects consisted of chondroplasty (surgically smoothening the joint surface without damaging surrounding tissues), and arthroscopic lavages and debridement (removing debris from the defect); however, these treatments are now known to be only palliative.^[^ ^7^ ^]^ To propose a functional repair of the OC defects, instead of merely alleviating the symptoms, bone‐marrow stimulation techniques such as microfracture were subsequently developed. Microfracture consists in debriding the defect and perforating small holes in the subchondral bone to induce the invasion of progenitor stromal cells in the defect,^[^ ^8^ ^]^ thereby contributing to tissue repair. Although this technique temporarily improves joint functions, it often leads to the formation of fibrocartilaginous repair tissue, the deterioration of the subchondral bone, and functional loss in the long term.^[^ ^9^, ^10^ ^]^

To overcome these limitations, and based on the assumption that the insufficient cell content in articular cartilage is the reason why it cannot regenerate by itself, autologous chondrocyte implantation (ACI) was developed.^[^ ^11^ ^]^ It consists in harvesting the patient's healthy cartilage on a non‐load‐bearing site, isolating and expanding its chondrocytes in vitro, and re‐implanting them in the defect, using a periosteal flap to maintain the implanted chondrocytes in place. This procedure has proven efficient in some patients with large cartilage defects (between 1 and 15 cm^2^)^[^ ^12^ ^]^ as well as OC defects;^[^ ^13^ ^]^ however, it requires two surgical procedures, a long recovery time, and is commonly associated with complications due to the hypertrophy of the flap.^[^ ^14^ ^]^

To improve this strategy, the scientific community then turned to the emerging field of tissue engineering. This field aims to combine interdisciplinary knowledge with engineering principles to restore, maintain, or improve tissue functions by designing biological substitutes,^[^ ^15^ ^]^ which can be achieved by combining cells with biomaterials and/or bioactive molecules. In this context, matrix‐associated ACI (MACI) was proposed as an alternative to ACI, where the autologous chondrocytes are cultured in 3D matrices before being implanted in the defect.^[^ ^16^ ^]^ Several biomaterials have now reached the market for OC defect repair, including monolayer (e.g., TruFit, BST‐Cargel, Bioseed‐C, Collagraft) and bilayer (e.g., ChondroMimetic, MaioRegen, Agili‐C, OsseoFit plug) systems, and can be used as delivery vehicles for drug and/or cell delivery.^[^ ^17^ ^]^ However, even though these current clinical treatments have a positive impact on joint mobility and pain reduction, they only lead to the formation of fibrocartilaginous neotissues that are not completely functional, as their mechanical properties are inferior to those of native cartilage.^[^ ^18^ ^]^

Taking advantage of new progress in biomaterial and cell‐based tissue engineering, the possibility to combine different matrices, cell types, and bioactive molecules has opened new promising avenues for OC regeneration. In the past few years, particular efforts have been made to combine biological elements with biomaterials having suitable biomechanical properties and architectures. Long overlooked, the subchondral bone is now believed to be a pivotal tissue for articular cartilage repair.^[^ ^19^ ^]^ Therefore, multilayered materials aiming to regenerate both articular cartilage and the underlying subchondral bone have been the subject of flourishing research, with the hope of identifying better restorative or regenerative options for OC defects. The multitude of new systems currently under investigations, and the variability of pre‐clinical results, make it difficult to identify key elements for success. In this context, putting into perspective the tissue engineering strategies that have been proposed in recent years is needed to progress toward successful OC regeneration.

The aim of this work is to perform a systematic review of biomaterial‐assisted strategies that have been tested in OC defects between 2015 and 2021. Almost 300 articles have been put into perspective to provide an overview of the field. This analysis includes critical considerations regarding: i) the assessment methods of OC repair, with considerations of the animal models, analysis techniques, and scoring systems used; ii) the general strategies (e.g., with or without cells and/or bioactive molecules, number of layers); iii) the chosen biological elements (i.e., cells and/or bioactive molecules); iv) the types and combinations of biomaterials; and v) the clinical relevance of the reported tissue engineering approaches. Based on this careful analysis and a biological understanding of the OC unit, we will discuss promising directions to address OC defect treatment, which still remains one of the most challenging clinical issues in orthopedics and rheumatology, notably because OC defects are one of the major risk factors of osteoarthritis.

## 2 An Overview of the Biology of the Osteochondral Unit

**Figure 1:**
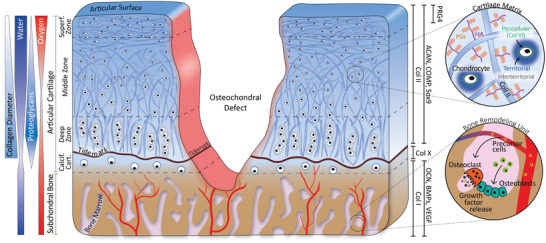
Schematic cross‐sectional representation of the OC unit presenting an ICRS grade IV OC defect. The different regions (subchondral bone, calcified cartilage, deep zone, middle zone, and superficial zone) and gradients of the unit are presented on the left. The main markers of the different zones are presented on the right. These include collagen (Col) types II and X, aggrecan (ACAN), cartilage oligomeric matrix protein (COMP), Sox9 and proteoglycan 4 (PRG4) for articular cartilage; and Col I, osteocalcin (OCN), Bone Morphogenic Proteins (BMPs), and vascular endothelial growth factor (VEGF) for the subchondral bone. The two circles are enlarged views of the cartilaginous and bone tissues. In cartilage, chondrocytes are embedded in a matrix mainly composed of Col II, hyaluronic acid (HA), and proteoglycans (PGs). In the subchondral bone, osteoblasts and osteoclasts remodel the matrix.

The OC unit is a complex structure with a hierarchical organization that relies on mechanical and biological integrity to function properly. It comprises two main tissues: the subchondral bone and the articular cartilage (**Figure** **1**). The latter can be further divided into four zones from the surface to the depth; namely, the superficial zone, the middle zone, the deep zone, and the calcified zone.^[^ ^1^ ^]^

Bone is a mineralized connective tissue, continuously remodeled by the catabolic activity of osteoclasts and the anabolic activity of osteoblasts, two of the cell types present in bones, giving it strong regenerative properties. It consists of both an organic and an inorganic phase. The organic phase is principally composed of collagen type I (Col I), as well as non‐collagenous proteins such as osteocalcin (OCN).^[^ ^20^ ^]^ The Col I fibrils are arranged into a porous structure, stabilized by intermolecular bonds, which partly contribute to bone yield strength.^[^ ^21^ ^]^ The inorganic phase is composed mainly of calcium and phosphate ions that precipitate to form biological apatite crystals. Together, the organic and inorganic phases form a composite structure that confers bone the major part of its mechanical properties. In the OC unit, the subchondral bone has a major role in the transmission of constraints. Its irregular surface, which secures cartilage anchoring, together with its mechanical properties, allows it to attenuate 30% of load forces, 1 to 3% being attenuated by cartilage, and the rest by the bone and the joint capsule.^[^ ^19^ ^]^ The subchondral bone is also essential due to its highly vascularized nature. In particular, tiny branches of its blood vessels extend to the calcified cartilage region, allowing metabolic exchanges at the interface between these two tissues,^[^ ^22^ ^]^ and ensuring an integrated response to chemical and mechanical stimuli.^[^ ^23^ ^]^ Finally, the subchondral bone also contains progenitor mesenchymal stromal/stem cells (MSCs) residing in the bone marrow, and can thus serve as a reservoir of progenitor cells when the OC unit is damaged.

Adult cartilage, on the other hand, is characterized by a low density of cells. Although progenitor cells have been found in a very limited number,^[^ ^24^, ^25^ ^]^ chondrocytes represent the vast majority of cartilage cells and occupy 5 to 10% of the tissue volume.^[^ ^26^ ^]^ Chondrocytes are responsible for the synthesis and the homeostasis of the ECM. Both the chondrocyte morphology and the ECM composition vary between the four cartilage regions. The superficial zone consists of an acellular protective layer of collagen, covering a layer of inert flattened fibroblast‐like chondrocytes.^[^ ^1^ ^]^ The ECM in this region is highly hydrated (75–80%), and mainly composed of Col II fibers that are oriented parallel to the articular surface, providing high shear resistance.^[^ ^27^ ^]^ From the superficial zone to the middle zone, the collagen fibers orientation switches from parallel relative to the articular surface to random; and from the middle to the deep zone, their orientation switches again from random to perpendicular relative to the articular surface. This particular organization, together with an increased collagen fiber diameter, is responsible for the tensile resilience and strength of cartilage.^[^ ^28^ ^]^ The other main components of the cartilaginous ECM are the proteoglycans (PGs). They are composed of negatively‐charged polysaccharides, namely glycosaminoglycans (GAGs), covalently linked to a protein core.^[^ ^29^ ^]^ These PGs are attached along hyaluronic acid (HA) chains, forming aggregates that are intertwined with collagen fibrils. Like the collagen content, the PG content varies along the depth of cartilage, as well as with the distance from chondrocytes. PGs are secreted in the pericellular matrix that immediately surrounds the cells (within 2 µm), and aggregate in the more distant interterritorial and territorial matrix to form aggrecan (ACAN).^[^ ^30^ ^]^ Together, due to their anionic nature, PGs and HA retain water, conferring cartilage a high resistance to compression.^[^ ^28^ ^]^ This hydrostatic pressure accumulates in the deep region, which is separated from the underlying calcified zone by a structure called the tidemark whose composition and supportive function are still a subject of debate.^[^ ^31^, ^32^ ^]^ The calcified cartilage has a composition different from that of the upper layers, comprising Col X and calcium phosphate (CaP) crystals, which results in intermediate mechanical properties—stiffer than non‐calcified cartilage, but softer than bone. This allows it to reduce stress concentrations at the interface between the two main tissues of the OC unit.^[^ ^33^ ^]^

Upon injury, bone can self‐repair to a certain extent, owing to its permanent remodeling, vascularized nature, and direct access to various sources of MSCs (i.e., periosteum, endosteum, and marrow cavity).^[^ ^34^, ^35^ ^]^ On the contrary, articular cartilage has a very limited capacity for self‐repair. This cartilage characteristic is commonly attributed to the extremely limited number of endogenous progenitors, and to the dense, avascular, aneural, and alymphatic nature of the tissue that is unfavorable for exogenous progenitor invasion and differentiation. Beyond these traditional considerations, the lifetime of cartilage ECM components has also been put into question. Contrary to GAGs that renew quite rapidly (half‐life between 300 and 800 days in humans, depending on the type of joint),^[^ ^36^ ^]^ the turn‐over of Col II is very low (half‐life of 117 years).^[^ ^37^ ^]^ This could justify the fact that mature individuals are incapable of restoring an efficient Col II network, which is essential for proper cartilage functioning.^[^ ^38^ ^]^ For all of these reasons, when the OC unit is damaged, the entire unit cannot fully regenerate; and no conventional treatment has been able to address this challenge. Therefore, innovative tissue engineering strategies have attracted increasing attention.

## 3 Evaluation Tools of OC Regeneration

One of the major challenges in the field of OC regeneration is to restore a functional OC unit rather than just filling the defect with a transient and unfunctional fibrocartilage. To assess whether the envisioned strategies are promising, it is thus crucial to properly evaluate the outcomes of potential new treatments in clinically relevant preclinical models, with comprehensive and comparable analyses. These should include careful observations of the repair tissues, both at the macroscopic and histological levels, as well as functional evaluations.

### 3.1 Animal Models

**Figure 2:**
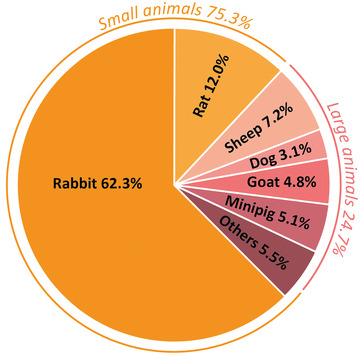
Animal models used for the evaluation of treatments for OC regeneration.

While a large body of relevant data has been generated from in vitro and ex vivo models, the restoration of the OC unit and of joint functionality cannot be fully evaluated without pre‐clinical in vivo models. When selecting an animal model for OC repair evaluation, many characteristics should be considered, including the age and maturity of the skeleton, the thickness of cartilage that is dependent on the size of the joint, the level of natural mechanical loading, the defect accessibility, and the ease of animal handling, as outlined in a recent review on animal models for OC regeneration assessment.^[^ ^39^ ^]^ To date, the vast majority of in vivo studies on OC repair tested their therapeutic approach exclusively in small animal models, using mainly rabbits and rats (**Figure** **2**), which could be explained by the cost and housing constraints‐related limited access to large animals.^[^ ^40^ ^]^ However, due to their small size, rodents present limited cartilage thickness, small defect volume, and their joints are subjected to lower loads than those of humans, which limits the evaluation of new strategies for potential translation to humans.^[^ ^41^ ^]^ Furthermore, rabbits, which is the most commonly used model, are known for their high endogenous OC self‐healing potential, which can easily create a bias in result interpretation.^[^ ^42^ ^]^ Only a few studies had access to large animal models, including sheep, goats, minipigs, dogs and horses. The anatomical characteristics (e.g., size of the joint, cartilage thickness) of these large animals are closer to those of humans,^[^ ^43^ ^]^ allowing a better assessment of implant performances. However, these models imply more logistical, financial, and ethical considerations.^[^ ^40^ ^]^

### 3.2 Analysis Methods

**Figure 3:**
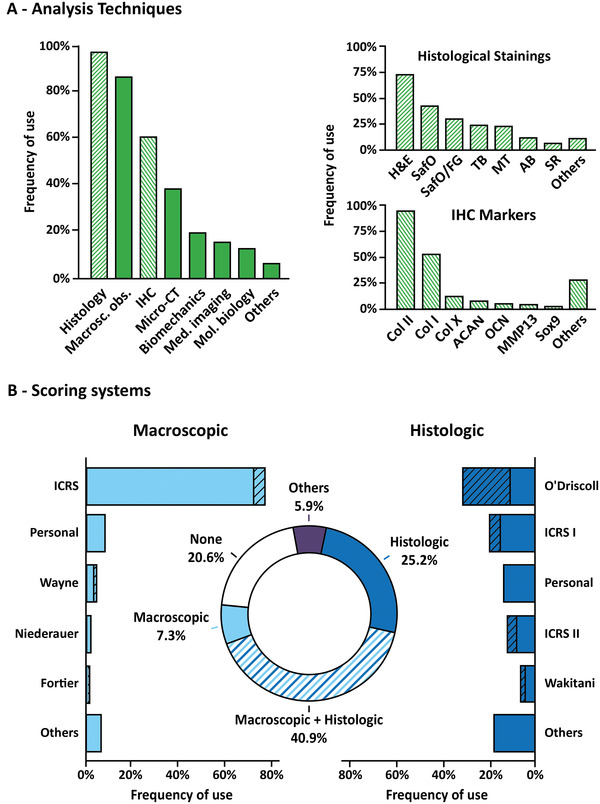
Evaluation tools of OC regeneration. A) Analysis techniques used to assess OC regeneration. The left panel represents the general techniques used, including histology, macroscopic observation (macro. obs.), Immunohistochemistry (IHC), micro‐computed tomography (Micro‐CT), biomechanics, medical imaging (Med. imaging), molecular biology (Mol. biology), and others. The hatched bars indicate the link with the right panels that represent in detail the different types of histological staining performed (top panel) and the different markers of articular cartilage and subchondral bone evidenced by IHC. HE: hematoxylin & eosin; SafO: Safranin O; SafO/FG: Safranin O counterstained with Fast Green; TB: Toluidin Blue; MT: Masson's Trichrome; AB: Alcian Blue; SR: Sirius Red; Col: collagen types I, II, and X; ACAN: Agreccan; OCN: osteocalcin; MMP13: Matrix Metalloproteinase 13. B) Scoring systems. The graph in the center represents the types of scores used in general. The left graph represents the macroscopic scores in detail. The right graph represents the histological scores in detail. The hatched portions represent the modified scores.

To determine whether strategies used to regenerate OC defects are effective, cartilage and subchondral bone repair must be assessed by various techniques, including the evaluation of the macroscopic aspect of the joint, the histological organization of the repaired tissues and their functional properties (**Figure** **3**). Regarding the macroscopic aspect of the joint, satisfactory results include a smooth surface, presenting the same color as the adjacent healthy cartilage, without a clear delimitation between regenerated and native tissues. Surprisingly, this relatively easy macroscopic assessment of the treated joints is not always evaluated (87%). On the other hand, histological staining, which consists of coloring tissues to assess their organization and to reveal the presence of certain markers, is performed almost systematically (Figure 3A).

For OC regeneration, satisfactory histological results correspond to newly formed bone and cartilage tissues showing tissue composition and organization that are as similar as possible to those of a native OC tissue (Figure 1). Thus, it is important to evaluate i) the expression of GAGs/PGs and collagens in proper locations, ii) the collagen fibers orientation, iii) the morphology and organization of the various chondrocyte phenotypes, including the observation of chondrocytes that are embedded in lacunae in the deep layer of cartilage, iv) the organization and mineralization of subchondral bone tissues, and v) the formation of a tidemark that separates subchondral bone and cartilage. Interestingly, our systematic review revealed that most studies only assessed cartilage quality by revealing the presence of GAGs/PGs, typically using Safranin O staining, Toluidine Blue, or Alcian Blue. Safranin O staining is often combined with Fast Green to evidence bone formation more easily. Collagen fibers are less commonly stained, either by Masson's Trichrome or Sirius Red. Surprisingly, although several colorations allow evaluating bone formation and mineralization, such as Movat's pentachrome or Goldner's Masson trichrome, they are rarely used for OC regeneration assessment.

To further histologically characterize newly formed tissues, immunohistochemistry analyses (IHC), where antibodies are used to evidence the presence of specific molecules, should be conducted, especially to detect the presence of the different collagen types, and assess whether the regenerated cartilage is hyaline‐like or undesirably fibrous. Our work showed that most studies only assessed cartilage markers, mainly investigating Col II as the main component of hyaline cartilage. The Col I content, a marker of undesirable fibrocartilage formation, is only studied in half of the publications using IHC; and aggrecan and Sox9 expression are less commonly analyzed. Interestingly, very few studies have investigated the expression of calcified cartilage and subchondral bone markers, such as Col X and OCN, which highlights the fact that the subchondral bone evaluation is under‐considered in OC repair. This is also confirmed by the uncommon use of microcomputed tomography (micro‐CT), which is a non‐destructive and non‐invasive method to evaluate mineralized tissues. It is noteworthy to mention that micro‐CT contrast agents now allow the assessment of cartilaginous tissues as well, making it a potential key technique for future OC regeneration evaluation.

Finally, OC repair assessment should also include an evaluation of the functional characteristics (e.g., biomechanical properties, lubrication properties) of the regenerated tissues, which is yet very rarely performed (less than 20% of the recent publications according to our analysis). Functional assessment is crucial because satisfactory joint mechanics is the ultimate goal of OC regeneration, and because implants often fail to restore proper biomechanical properties even when macroscopic and histological outcomes are encouraging. As emphasized in a recent review on mechanical testing of articular cartilage,^[^ ^44^ ^]^ compression tests should be systematically performed to determine the stiffness and relaxation properties of the newly formed tissues, as well as nanoindentation tests to characterize these tissues at the microscopic scale (e.g., stiffness gradients within different regions of the OC unit). Ideally, tissue integration, including bottom and lateral anchoring, should also be characterized by push‐out or shear tests; and lubrication should be evaluated.

### 3.3 Scores

Different scoring systems have been developed for OC repair assessment, allowing to systematically and statistically evaluate treatment outcomes, and put into perspective the qualitative examinations of macroscopic aspect, histology, and medical imaging. These scoring systems can be applied for macroscopic assessment and histological assessment (Figure 3B). Although different scoring systems exist for macroscopic evaluation, there seems to be a consensus, with more than half of the studies since 2015 using the ICRS score or its variations, as evidenced by our systematic review. This score assesses the overall appearance of the repaired tissue in the most easy and relevant way, based on observations regarding the degree of defect repair (% repair of defect depth), the integration to the border zone (presence or absence of a demarcating border), and the macroscopic appearance (smoothness of the surface). It is noteworthy that the color of the neo‐formed cartilage is relevant information that is not considered in the ICRS score. This is important, as yellow or brownish tissues are generally a sign of undesirable fibrocartilage formation. Thus, a new scoring method that includes this parameter has been proposed by Wayne et al.,^[^ ^45^ ^]^ and used by several authors since then, under the name of “Wayne score”.

For histological assessment, a plethora of scoring systems has been used, surprisingly including some that aim to assess osteoarthritis progression, which is debatable in the context of OC repair. Orth and Madry provided an overview of the different histological scores for in vivo cartilage repair assessment.^[^ ^46^ ^]^ Ideally, histological scoring should consider both the general appearance of the OC unit (e.g., smoothness of the surface, homogeneity of the regenerated tissues), and also the composition, the thickness and integration of the newly formed ECM, the distribution and morphology of the cells in the different regions of the OC unit, the formation of a new tidemark, the OC junction, along with the subchondral bone regeneration. To date, there is no established score that fully takes all of these characteristics into account. Thus, many modified scores have been used, where the authors added to established scoring systems what they consider to be important criteria, such as the modified O'Driscoll or the modified Wakitani scoring systems.^[^ ^48^, ^49^ ^]^ However, modifying a score could be a source of biases and should be cautiously considered. Overall, the variety of scoring methods leads to a huge disparity in tissue evaluations and data interpretation, making the comparison between studies very difficult, if not impossible. Future strategies for OC repair will thus have to be evaluated according to a consensual scoring system. According to us, the Sellers score^[^ ^50^ ^]^ constitutes a potential candidate to reach a scoring consensus. This score considers common parameters for the evaluation of OC regeneration, that is, the filling of the defect, the integration with the surrounding tissues, the composition, the cellular morphology, and the architecture of the surface. More importantly, it is the only score that includes the following key elements: the architecture of the tissues within the defect, the subchondral bone formation, and the tidemark formation.

## 4 OC Regeneration Strategies: Overall Considerations

**Figure 4:**
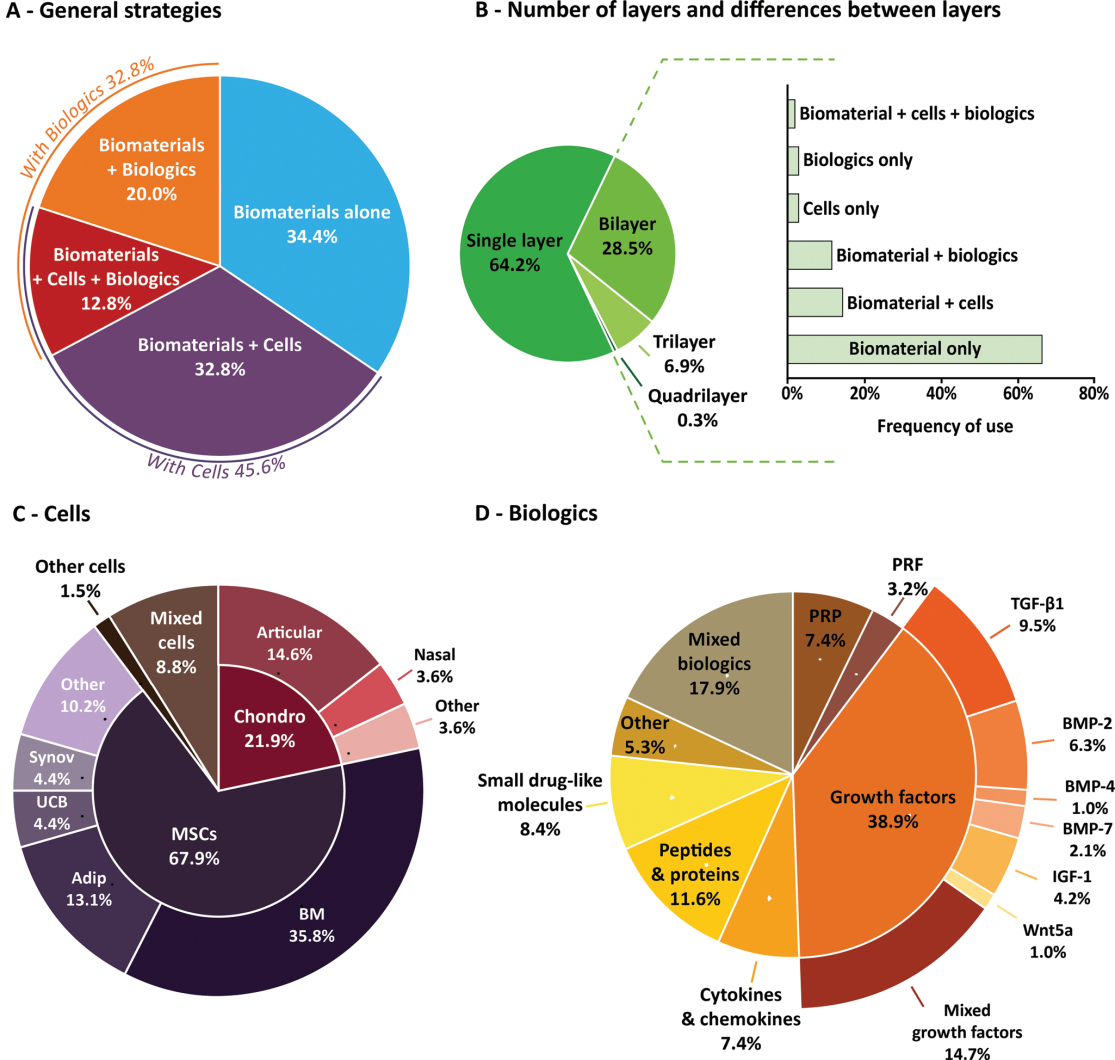
OC regeneration strategies. A) General strategies for OC regeneration including the use of a biomaterial alone or combined with cells and/or biologics. B) Number of layers and differences between the layers when multilayer materials are implanted. Biomaterial differences include differences in the composition and/or the structure of the material. C) Cell types used for OC regeneration: chondrocytes (Chondro), mesenchymal stromal/stem cells (MSCs) or MSC‐derived cells from the bone marrow (BM), adipose tissue (Adip), umbilical cord blood (UCB), synovial tissues (Synov) or other sources, as well as other cells or mixed cells. D) Analysis of bioactive molecules used in biologics‐based approaches, including platelet‐rich plasma (PRP), platelet‐rich fibrin (PRF), transforming growth factor‐ß1 (TGF‐ß1), bone morphogenetic proteins (BMP)‐2,‐4,‐7, insulin‐like growth factor‐1 (IGF‐1), wingless‐type family member 5a (Wnt5a), mixed growth factors, or other types of bioactive molecules.

OC restoration requires the challenging development of therapeutic approaches able to trigger regenerative mechanisms that are not naturally present in adult cartilage. To succeed, researchers have investigated the use and combination of large varieties of cells, biologics, and biomaterials (**Figure** **4**). Traditionally, the lack of regeneration was attributed to an insufficient cell density in articular cartilage, encouraging the development of cell‐based therapies. In this context, biomaterials were mainly used to deliver cells and maintain them in the defect, while biologics (i.e., growth, systemic, and/or differentiation factors) were added to control cell fate. The disappointing results of these early strategies in humans came at a time when pioneering studies demonstrated the importance of cell‐material interactions for biological functions.^[^ ^51^, ^52^, ^53^, ^54^ ^]^ It paved the way for a new era of research, where biomaterials are designed with appropriate structures and biomechanical properties to improve cell functions. In parallel to cell therapy enhancement, a great body of work has focused on the design of materials that can themselves (without cells or biologics) promote OC repair. It is now commonly admitted that biomaterial scaffolds can play a key role in innovative OC defect therapy, allowing to i) physically fill a defect, ii) control the local delivery of therapeutic agents, and iii) guide regeneration.

Thus, while cellular strategies were largely predominant a few years ago (65% of the investigated strategies according to a review published in 2015),^[^ ^55^ ^]^ research seems to evolve toward acellular approaches, which represent less than half (46%) of the investigated systems since 2015, as revealed by our systematic review (Figure 4A). Among these systems, multilayered implants are more and more frequently considered as a step toward better OC tissue reconstruction (Figure 4B).

### 4.1 Toward Multilayered Implants

To regenerate an OC unit, the restoration of both the subchondral bone and cartilage tissues must be addressed, notably to reach significant clinical benefits for the patients. The feasibility of bone repair has long been demonstrated, and can be achieved with acellular strategies, using one of the numerous bone substitutes (e.g., animal or human bone, polymers, ceramics, composite materials) now commercially available.^[^ ^56^ ^]^ Furthermore, bone exhibits natural regeneration capacities, making small bone defects overall relatively easy to repair.^[^ ^57^ ^]^ Conversely, cartilage regeneration remains a challenge to date, requiring the reconstruction of complex ECM and cell organizations in three dimensions, with specific compositions and biomechanical properties. For these reasons, until very recently, research on OC regeneration focused almost exclusively on cartilage repair rather than addressing the entire OC unit; and most of the reported strategies made use of implants composed of a single phase filling the entire defect (64%). However, the potential of success of strategies that solely address cartilage repair is more and more questioned because the proper subchondral bone repair is required to allow cartilage regeneration.^[^ ^58^ ^]^

The optimal regeneration of both tissues can hardly be addressed by a single‐composition strategy because the subchondral bone and cartilage are two very different tissues: the former is stiff (varying from 1.6 to 3.9 GPa depending on the joint),^[^ ^59^ ^]^ porous, vascularized, and rich in metabolically active cells; while the latter is relatively soft (0.1 to 6.2 MPa),^[^ ^33^ ^]^ with low oxygen and nutrient supplies, and with a low density of cells that have a limited metabolic activity. This observation calls for the combination of two distinct biomaterial‐assisted strategies, and led to the recent development of new bilayer implants. The relevance of this approach has been demonstrated by several studies reporting better outcomes with bilayer implants compared to single‐layer implants in different animal models.^[^ ^60^, ^61^ ^]^ To further mimic the OC structure, several research teams have designed materials with an additional transition layer that has intermediate compositions and mechanical properties to improve the transmission of constraints between tissues, which is a key feature of calcified cartilage. For example, a trilayer structure implanted in a goat model of OC defects, in both load‐bearing and non‐load‐bearing sites, convincingly led to the successful regeneration of the entire OC unit, with the additional restoration of the tidemark.^[^ ^62^, ^63^ ^]^ This study, however, did not include the corresponding bilayer implant as a control, but a commercial bilayer implant instead, which renders the benefit of the third layer difficult to assert.

Besides its mechanical role, the calcified cartilage also acts as a physical barrier between bone and cartilage, inhibiting blood vessel invasion from the subchondral bone and the subsequent ossification due to osteogenic signals,^[^ ^64^ ^]^ and also inhibiting cartilage invasion in the subchondral bone. Thus, the use of an isolating third layer was also envisioned. In a recent study, both a trilayer and the corresponding bilayer implant without an isolating layer were implanted in OC defects of the medial femoral condyle of goats.^[^ ^65^ ^]^ After 6 months, porcelain white cartilage and subchondral bone almost indistinguishable from the host bone were obtained with the trilayer implant, whereas yellow fibrocartilage invading a non‐calcified cancellous subchondral bone tissue was observed with the bilayer implant, showing the potential efficacy of such a strategy. Another study reported the use of a separating layer to maintain the upper layer avascular and with a low oxygen tension, which promotes chondrogenic differentiation of MSCs.^[^ ^66^ ^]^ 48 weeks after implantation in OC defects in the medial femoral condyle of goats, no difference was observed between the trilayer implant and bilayer control, suggesting a limited contribution of the insulating layer. However, both systems showed promising results in terms of macroscopic, histological, and biomechanical outcomes.

Interestingly, our work indicates that the vast majority of the investigated multilayered implants focused on the sole adjustment of the biomaterial used in each layer, highlighting the potential for new investigations. Overall, multilayer implants are receiving growing attention as a second‐generation approach for material‐assisted OC repair, but their more complex design and only recent development do not position them yet as a clear strategy of choice in the community.

### 4.2 Importance of Biological Elements

Facing the limitation of biomaterials alone in terms of repair efficacy, their association with biologics has been considered. Indeed, the successful regeneration of the OC unit may greatly depend on the presence of biological elements, that is, cells and bioactive molecules. In particular, growth factors (GFs) that can trigger physiological events (e.g., development, growth) could play an important role. In this context, finding the optimal combination of biological elements may be a key for success. Considering the tissue differences between bone and cartilage, different strategies have been developed to optimize the regeneration of each of the two tissues.

#### 4.2.1 Cells

The ultimate goal of OC tissue engineering is to restore a tissue with the same cell types organized in the same way and with the same functions as the healthy tissue. To reach this goal, the preclinical autologous, allogenic, or xenogeneic transplantation of cells has also been considered an attractive approach, as they can secrete the specific ECM of a given tissue. This potential to recreate adequate ECM is one of the major criteria for cell type selection, along with their accessibility. Based on these considerations, three main strategies have been used for OC regeneration, namely the delivery of (i) cells naturally presents in joint tissues, (ii) MSCs, and (iii) MSC‐derived osteogenic or chondrogenic cells, along with diverse combinations (Figure 4C).

Cells specifically found in the OC tissue, and that can synthetize ECM, include osteoblasts for the subchondral bone, and chondrocytes for articular cartilage.^[^ ^67^ ^]^ Chondrocytes have been used in the clinic for years (ACI, MACI), and seem to be an obvious choice for the regeneration of the cartilage compartment since they are capable of synthetizing and remodeling its specific ECM. However, primary chondrocytes have several drawbacks. First, they can only be collected in small quantities,^[^ ^30^ ^]^ and their harvesting is associated with morbidity and cartilage degradation at the donor site.^[^ ^68^ ^]^ Second, chondrocytes tend to de‐differentiate during in vitro expansion, as illustrated by a switch from the production of Col II (specific to articular cartilage) to that of Col I (found in fibrocartilage).^[^ ^69^, ^70^ ^]^ This phenotype instability is associated with unwanted replicative senescence.^[^ ^71^ ^]^ An alternative to autologous cartilage harvesting is the use of chondrocytes from allogenic or xenogeneic origins, that is, coming from a different individual or from a different species, respectively. Although it avoids problems associated with donor site morbidity and cell quantity, it can induce immune responses that gradually destroy the treated cartilage,^[^ ^72^ ^]^ and increases the risk of disease transmission.

Because of these limitations, the use of MSCs or osteochondrogenic cells has been envisioned to regenerate both the subchondral bone and the cartilage compartments of the OC unit. Initially discovered in the bone marrow,^[^ ^73^, ^74^ ^]^ MSCs are defined as multipotent cells that can self‐renew. They can differentiate into various cell types including osteoblasts and chondrocytes,^[^ ^75^ ^]^ which is especially relevant for OC repair. MSCs can be found in a variety of vertebrate tissues and fluids, including peripheral blood and adipose tissue, as well as in umbilical cord blood and synovial membrane. They are fairly easy to harvest, and have a high in vitro expansion capacity, making them an interesting cell source.^[^ ^76^, ^77^, ^78^ ^]^

Among MSCs, bone marrow‐derived MSCs (BMSCs) are relatively easy to harvest and have high osteochondrogenic potential, making them cells of choice for OC regeneration. Many studies have thus investigated the use of BMSCs combined with biomaterials for OC repair.^[^ ^79^, ^80^, ^81^ ^]^ Yet, their harvesting induces morbidity and pain at the donor site, and more importantly, these cells are only available in limited quantities. Adipose‐derived MSCs (ASCs) constitute an attractive alternative to BMSCs, as they are more abundant and accessible.^[^ ^82^, ^83^ ^]^ It is worth mentioning that a study suggested encapsulated autologous ASCs were not as effective as encapsulated autologous BMSCs or cartilage progenitor cells for OC defect regeneration in an ovine model, as evidenced by poorer outcomes (irregular articular surface with the presence of fibrous tissues in some areas).^[^ ^84^ ^]^ However, this study showed a high inter‐individual variability that calls for further work and corroborative studies.

Among the other cell sources available, synovial MSCs (synMSCs) are attractive for OC regeneration because they can be harvested from the synovial fluid in a minimally‐invasive manner, and have a high capacity for proliferation and chondrogenic differentiation compared to BMSCs;^[^ ^85^, ^86^ ^]^ yet, they have rarely been investigated.^[^ ^87^, ^88^, ^89^ ^]^ Also, a comparative study showed that SynMSCs have a lower regenerative potential than chondrocytes in a rabbit OC defect model, forming a tissue with a lower biomechanical strength and a lower histological score.^[^ ^90^ ^]^ Surprisingly, while pluripotent stem cells (including induced or embryonic stem cells) have been described as promising for cartilage^[^ ^91^, ^92^, ^93^ ^]^ and bone regeneration,^[^ ^94^ ^]^ they have only marginally been tested in association with biomaterials for OC regeneration in the past few years.^[^ ^95^, ^96^ ^]^

Finally, a third approach consists in combining tissue‐specific cells with MSCs. Co‐cultures of MSCs (mainly BMSCs) and chondrocytes lead to mutual benefits, with favored MSC differentiation into chondrocytes, and the maintenance of proper chondrocyte phenotype.^[^ ^97^, ^98^ ^]^ Yet, in the context of OC regeneration, the combination of chondrocytes with MSCs in the cartilage layer has only been little studied.^[^ ^99^, ^100^ ^]^

Although MSCs and progenitor cells overcome the difficulties (harvesting, quantities) associated with the native cells of the OC unit, their heterogeneity, as well as their variable differentiation potential still limit their use.^[^ ^101^ ^]^ Beyond the cell source, cell fate is greatly influenced by a multitude of microenvironmental parameters, especially by signaling molecules. Thus, in addition to maintaining an appropriate cell type in an optimal biomaterial, OC regeneration may also require the use of bioactive molecules involved in specific differentiation pathways. The following section focuses on bioactive factors that influence the recruitment, differentiation, or maintenance of cell phenotype to promote OC regeneration.

#### 4.2.2 Bioactive Molecules

Bioactive molecules can be combined with biomaterials by several methods, for example, by adding them to the precursor solutions, by impregnating a scaffold with a solution containing the biologics, by integrating the molecules to the polymer network, or by encapsulating the biologics in micro‐ or nanospheres. These molecules are mainly GFs, small drug‐like molecules or cytokines, involved in regulating the development and growth of subchondral bone and articular cartilage (Figure 4D).

GFs are key biological signaling elements for tissue development, growth, homeostasis, and regeneration, and have therefore been extensively studied in the context of OC tissue engineering (39% of the systems reported since 2015). In particular, the members of the transforming growth factor‐*β* (TGF‐*β*) super‐family have attracted much attention for both bone and cartilage as they play critical roles in their development.^[^ ^102^ ^]^ More specifically, in the context of OC regeneration, TGF‐*β*1, TGF‐*β*3, and three of the bone morphogenetic proteins (BMPs) large subfamily (BMP‐2, BMP‐4, and BMP‐7) have widely been used.^[^ ^103^ ^]^ Their effects on cell proliferation and differentiation into osteoblasts or chondrocytes are time‐dependent, dose‐dependent, and context‐dependent.

The expression of TGF‐*β*1, TGF‐*β*3, and their receptors varies between the different zones of the OC unit, and during growth.^[^ ^104^ ^]^ A high expression of TGF‐*β* receptors is found on osteoblasts, and the highest expression of TGF‐*β*1 and TGF‐*β*3 is in bone during growth, where they i) promote the differentiation of osteogenic progenitors, ii) stimulate matrix production, and iii) inhibit osteoblast maturation, mineralization, and transition into osteocytes.^[^ ^105^ ^]^ They are also well acknowledged to induce the chondrogenic differentiation of MSCs and promote the synthesis of PGs and Col II by chondrocytes.^[^ ^106^ ^]^ These interesting effects drove researchers to use these factors to enhance OC regeneration. For example, TGF‐*β*1 was loaded in the upper layer of a temperature‐responsive bilayer material, which enhanced the chondrogenic differentiation of human BMSCs in vitro compared to the control, and promoted cartilage regeneration (tissue composed of GAGs and Col II) in a rat model of OC defect.^[^ ^107^ ^]^

BMPs also have an essential role in osteogenesis and chondrogenesis during skeletal morphogenesis. They have proliferative effects, chemotactic properties, and can induce MSC differentiation toward both osteoblastic and chondrogenic lineages,^[^ ^108^ ^]^ depending on the cellular microenvironment and the interaction with other regulatory factors.^[^ ^109^ ^]^ In cartilage, BMPs (in particular, BMP‐2, ‐4, and ‐7) promote chondrocyte differentiation and can enhance their Col II and ACAN production.^[^ ^106^ ^]^ BMP‐2, a Food and Drug Administration (FDA)‐approved osteoinductive and chondrogenic GF, is the most frequently used member of the BMP family. Yet, its adverse effects are well‐known (e.g., ectopic bone formation, bone cyst formation), and attributed to burst release and the local delivery of high doses of BMPs.^[^ ^110^ ^]^ It is thus crucial to control BMP delivery to achieve proper OC repair. This challenge has been addressed in a recent preclinical safety study, where an implantable scaffold (ARTiCAR) was designed to locally and sustainably release BMP‐2 to physiological levels. The device has shown encouraging results in sheep, with assessment scores higher than those of the control group.^[^ ^111^ ^]^

Other than GFs, small drug‐like molecules have been used for OC regeneration, the most investigated one being kartogenin.^[^ ^112^ ^]^ This factor is gaining increasing attention as it was shown to stimulate the chondrogenic differentiation of MSCs, prevent cartilage degeneration, and subchondral bone degradation.^[^ ^113^ ^]^ In the context of OC regeneration, this molecule has shown potential as a cell‐homing molecule,^[^ ^114^ ^]^ that is, a molecule capable of attracting local MSCs. This property is interesting for the development of cell‐free systems that can circumvent the limitations associated with cell therapy, including the expensive and time‐consuming steps of cell harvesting and in vitro expansion. Kartogenin has been increasingly used alone^[^ ^115^ ^]^ or combined with other molecules.^[^ ^116^ ^]^ In particular, kartogenin was studied in combination with a newly designed high‐affinity oligonucleotide (aptamer A19S) that specifically binds to human stem cells.^[^ ^117^ ^]^ This led to more BMSC migration and binding to the scaffold in vitro compared to a scaffold without aptamers, and allowed the better repair of rat OC defects, evidenced by stronger GAGs and Col II staining, and a higher percentage of subchondral bone formation.^[^ ^118^ ^]^

Although the addition of a single bioactive factor to the delivered system seems to be promising, there is a multitude of factors involved in the complex processes of OC biological development and homeostasis. Thus, the administration of multiple factors to further enhance the regenerative potential of treatments may be necessary, and is gaining attention. For example, using a protein scaffold, the co‐delivery of TGF‐*β*1 and insulin growth factor (IGF)‐1, a circulating cytokine that plays a role in cartilage homeostasis and prevents joint degeneration, was shown to allow complete bone healing and successful cartilage regeneration.^[^ ^119^, ^120^ ^]^ Going one step further, platelet‐rich plasma (PRP) and platelet‐rich fibrin, which contain a plethora of GFs as well as other bioactive molecules, have been investigated. The addition of PRP greatly enhanced the healing potential in several studies.^[^ ^121^, ^122^, ^123^ ^]^ However, the undefined and variable composition of PRP is a major limitation that can lead to an important disparity in outcomes;^[^ ^124^ ^]^ and its benefits are most often reported without a clear understanding of the underlying mechanisms. Therefore, the growing interest for PRP in OC defects treatment should be observed with caution; and identifying adequate combinations of GFs would be preferable to further advance the field.

During biological development and healing processes, all of the factors involved intervening in a local and sequential way. It is therefore important to control the delivery of bioactive factors in a spatiotemporal manner. Yet, less than half of the studies involving bioactive factors claim to achieve controlled or sustained release (data not shown), with most of the work on sustained‐release only.^[^ ^116^, ^125^, ^126^ ^]^ A recent study reported the development of a three‐layer biomimetic biomaterial that allows the local and sustained co‐delivery of BMP‐2 in the bone layer and that of TGF‐*β*3 in the chondral layer.^[^ ^127^ ^]^ The slow and prolonged GF release over 30 days led to the regeneration of OC defects at 16 weeks in a rabbit model, with a trabeculae‐presenting subchondral bone, and proper hyaline‐like cartilage formation (i.e., smooth surface, abundant GAG and Col II content, some chondrocytes embedded within lacunae). To better recapitulate biological processes, the sequential release of multiple factors is also of great interest. For example, the initial release (within 24 h) of SDF‐1 that induces cell migration into the defect, followed by the release (over 3 days) of a small molecular drug (Y27632—a chondrogenic molecule) that induces in situ cell differentiation into chondrocytes, was shown to improve GAG deposition compared to conditions without bioactive factors in a rabbit model.^[^ ^128^ ^]^

The direct delivery of GFs can be limited by their short half‐life after encapsulation, and by the adverse effects associated with burst release. An alternative to direct GF delivery is the use of gene therapy to regulate the long‐term expression of proteins of interest by host cells. For example, to overcome the limitations of free BMP‐7 delivery, a system in which BMSCs transfected with a lentivirus coding for the BMP‐7 gene was used to improve bone and cartilage regeneration.^[^ ^129^ ^]^ Twelve weeks after implantation in beagle dogs, the OC defects were completely filled with cartilage‐like tissue of the same thickness as surrounding tissues, and presented the most bone formation. Likewise, it has recently been shown that the release of a vector overexpressing the chondrogenic factor sox9 from an in situ‐forming temperature‐responsive hydrogel improved OC repair compared to the administration of an empty vector.^[^ ^130^ ^]^ This promising option is still largely under‐investigated in the context of OC defects treatment.

Although many efforts have been made, controlled delivery is certainly one of the main challenges of the field to achieve an adequate release of bioactive factors and, ultimately, direct cell fate. To this end, biomaterial design is gaining increasing attention.

### 4.3 Biomaterials for OC Regeneration

As aforementioned, the successful regeneration of an OC unit will most likely necessitate the combination of biomaterials designed specifically to regenerate each of the two tissues of interest: subchondral bone and cartilage.

#### 4.3.1 Biomaterials for Subchondral Bone Regeneration

**Figure 5:**
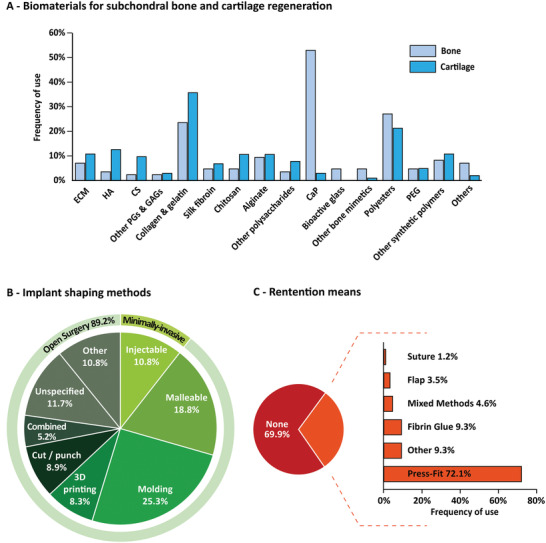
Composition, shaping, and retention of OC implants. A) Biomaterials for subchondral bone and cartilage regeneration, including extracellular matrix (ECM); hyaluronic acid (HA), chondroitin sulfate (CS), other proteoglycans (PGs) and glycosaminoglycans (GAGs), calcium phosphates (CaP), polyethylene glycol (PEG). Percentages represent the number of studies including each polymer in the biomaterial, either alone or in combination with others. B) Implant shaping methods. C) Retention means used to maintain the implant in the defect. Here, easy‐to‐handle materials that can fill the defect but that are not injectable through a needle or syringe are considered malleable.

To favor bone regeneration, ceramics, and in particular CaPs, have been extensively studied. CaPs have physicochemical similarities to the biological apatite that is naturally present in bones, giving them similar properties in terms of biodegradability, osteoconductivity and, in some cases, osteoinductivity. Osteoconductivity has been defined as the ability to promote bone formation on the surface of a material, while osteoinductivity is the inherent ability to induce bone formation in ectopic sites or from the center of the implant in orthotopic sites.^[^ ^131^, ^132^ ^]^ Osteoconductive properties of CaPs are mediated by the dissolution and reprecipitation of CaPs, incorporating other ions from physiological fluids to form carbonated apatite, which induces protein adsorption and thus cell attachment and differentiation. Among CaPs, hydroxyapatite (HAP) and tricalcium phosphate (TCP), which differ in their calcium/phosphate ratio and crystalline structure, are of particular interest because they can also present osteoinductive properties, depending on their composition, topography, and porosity.^[^ ^56^ ^]^ As TCP is more soluble than HAP, the two components can be combined in so‐called biphasic CaPs,^[^ ^133^ ^]^ using various ratios to tune the material resorption rate and match that of bone formation. Besides their biological performance, CaPs are easy to produce, inexpensive and safe, which is why they are commercially available and widely used in the clinic. For all of these reasons, CaPs are by far the most commonly used biomaterials (53%) for bone regeneration in the context of OC defects (**Figure** **5**).

Other ceramics for bone regeneration include bioactive glasses (BGs), which are defined as glasses that bond chemically to bone,^[^ ^134^, ^135^ ^]^ and comprise silicate, phosphate, or borate‐based glasses. Their excellent bioactivity is attributed to two mechanisms: i) an interaction between the local collagen fibrils and the surface of BGs, allowing them to bind strongly to bone; and ii) their progressive dissolution and reprecipitation in physiological fluids, which leads to the formation of hydroxycarbonate apatite and gives them osteoconductive properties.^[^ ^136^, ^137^ ^]^ The release of ions, and in particular that of cations, is also believed to support chondrogenesis,^[^ ^138^ ^]^ which is of interest in the context of OC regeneration. For example, a BG‐loaded alginate hydrogel seeded with BMSCs used as a bone layer, combined with a cartilage‐regenerating layer (composed of a hydrogel loaded with articular chondrocytes and BMSCs), was shown to improve OC repair compared to the bone layer alone and the cartilage layer alone in a rat model.^[^ ^100^ ^]^ In general, even though BGs have long been commercially available and have given excellent results for bone regeneration, they have not met the commercial success of CaPs, possibly because the compositions that are best suited for bone repair are difficult to process into scaffolds.^[^ ^139^ ^]^

Although ceramics are efficient in regenerating bone defects, their brittleness limits their use in load‐bearing areas. The field of bone tissue engineering has thus evolved toward the development of bone substitutes that combine bioactive ceramics with polymers to obtain more ductile and easy‐to‐handle materials with tunable properties (i.e., stiffness, fracture toughness, porosity, surface topography).^[^ ^140^ ^]^ For example, CaP suspensions in viscous solutions, and composite cements in the form of malleable viscous pastes that can harden in vivo, have been extensively developed as they both offer the possibility to treat bone defects with minimally‐invasive surgery, contrary to conventional ceramic blocks and granules.^[^ ^141^ ^]^ Currently, the challenge is to develop cements with macroporosity to improve their degradation rate and therefore facilitate bone regeneration.^[^ ^142^ ^]^ Advances in this field could be of great interest for OC regeneration, as better subchondral bone healing should allow better outcomes regarding cartilage repair.

#### 4.3.2 Biomaterials for Cartilage Regeneration

A large variety of biomaterials have been tested for cartilage regeneration, with the aim to mimic the natural composition and physicochemical properties of articular cartilage (Figure 5A). Among them, ECM‐based materials are an appealing choice as they contain all of the natural matrix components, properly organized in three dimensions,^[^ ^143^ ^]^ allowing them to influence cell fate and retain bioactive factors.^[^ ^144^ ^]^ In this context, the use of decellularized ECM from bone,^[^ ^129^ ^]^ articular cartilage,^[^ ^145^, ^146^ ^]^ as well as a whole decellularized OC unit,^[^ ^147^ ^]^ have been investigated for OC regeneration. Together, these studies have shown promising results, both in small and in large animal models. However, ECM should be taken either from the patient themselves, which is invasive, or from allogeneic or xenogeneic origin, which can cause immunologic rejection.^[^ ^148^ ^]^ As an alternative to decellularized ECM, the incorporation of ECM components (e.g., GAGs, collagens) is most often considered. It is now established that the ECM physicochemical properties, such as stiffness, architecture, and surface topography, influence the functions of cells.^[^ ^149^, ^150^ ^]^ For cartilage regeneration in particular, it is necessary to design scaffolds that recapitulate its high water content, composition, and viscoelastic properties. A great body of work has been dedicated to the development of hydrogels, which are hydrated polymer networks that best mimic ECM features.^[^ ^151^ ^]^ To do so, many polymers have been used and can be combined, including polysaccharides (e.g., HA,^[^ ^115^, ^152^ ^]^ alginate,^[^ ^118^, ^121^ ^]^ agarose,^[^ ^100^, ^153^ ^]^ chitosan,^[^ ^154^, ^155^, ^156^ ^]^ cellulose derivatives^[^ ^47^ ^]^), proteins (e.g., collagen,^[^ ^120^ ^]^ gelatin,^[^ ^157^, ^158^ ^]^ silk fibroin,^[^ ^159^ ^]^ fibrin,^[^ ^160^ ^]^), and synthetic polymers (e.g., polyethylene glycol (PEG)),^[^ ^161^ ^]^ reflecting the great number of possibilities, but also the lack of clear guidance in the matter.

Among the mechanical properties of hydrogels, stiffness was demonstrated to have a critical role, as MSCs express more osteogenic markers when cultured on stiff (15–40 kPa) versus soft (0.1–1 kPa) matrices.^[^ ^51^ ^]^ More recently, it was evidenced that MSCs were also sensitive to the viscoelastic properties of hydrogels, which reflect the matrix's ability to store or dissipate forces.^[^ ^162^ ^]^ The impact of such properties was recently investigated in the context of OC regeneration, using PEG/glycerol/sebacic‐acid‐based hydrogels with different stress relaxation properties.^[^ ^161^ ^]^ The authors demonstrated that the fastest‐relaxing hydrogel favored chondrogenesis in vitro, and further confirmed its regenerative potential in a rabbit model of OC defects, with the successful formation of well‐integrated articular cartilage 12 weeks post‐implantation. From a cell‐material interaction perspective, developing hydrogels with tailored mechanical and viscoelastic properties is thus a key element. Success with hydrogels has, however, been limited to date, mainly because they often lack sufficient mechanical properties to withstand the high and repeated mechanical loading to which natural articular cartilage is subjected.^[^ ^163^ ^]^ To address this challenge, fiber‐reinforced systems are used in tissue engineering, where a hydrogel is combined with rigid nano‐ or microfibers that are obtained via the electrospinning of synthetic polyesters such as polycaprolactone (PCL). This strategy has attracted little attention for OC regeneration to date,^[^ ^61^, ^81^ ^]^ leaving room for innovation.

Ideally, besides adequate mechanical properties, other parameters including the biodegradation profile and the porosity of biomaterials, should be considered. Biodegradability is an important parameter for tissue regeneration, as the scaffold should degrade to allow new ECM formation, but not degrade too fast to support cells and/or biologics for the necessary time. To control the scaffold degradation rate, one can use biodegradable polymers with tunable biodegradability, such as the synthetic poly(lactic‐co‐glycolic) acid (PLGA). PLGA is made of a ratio of polylactic acid (PLA) and polyglycolic acid (PGA) that can be modulated to adjust the complete degradation time of the copolymer from 10 to more than 35 weeks.^[^ ^164^ ^]^ Interestingly, PLGA also allows to design scaffolds with tunable porosity, which is a property playing an essential role in bone regeneration^[^ ^165^ ^]^ and that was shown to impact OC repair. In a first study, a series of bilayer PLGA implants varying in the pore size of their subchondral and chondral layers was prepared, and showed differences in repair potential 12 weeks after implantation in rabbit OC defects.^[^ ^166^ ^]^ The authors identified one combination of pore sizes (100–200 µm for the chondral layer, and 300–450 µm for the subchondral bone layer) that led to the best biological outcomes, and further confirmed the results in a longer in vivo study.^[^ ^167^ ^]^ Although this scaffold in combination with BMSCs led to the formation of tissues with much better histological scores than the untreated control, the mechanical properties of the repair tissue remained inferior to those of a healthy cartilage, calling for further investigations.

From all of these studies, it is evident that the design of an ideal biomaterial scaffold for OC regeneration remains a challenge, which will have to be addressed with great consideration regarding composition, mechanical and structural properties. Beyond material design, the clinical relevance of the delivery strategy must be considered for translation to human patients.

## 5 Clinical Relevance

The morbidity at the donor site, and the long recovery times needed after surgery, constitute two of the major drawbacks associated with current treatments such as ACI.^[^ ^14^ ^]^ The type of surgical intervention (i.e., a minimally‐invasive intervention or open surgery) is thus an important aspect of the treatment. It is conditioned by several factors including the type of biomaterial (i.e., injectable or not), the method used to shape it into an implant, and the method used to maintain the implant into the defect. All of these aspects should be considered when designing clinically relevant strategies for OC regeneration.

### 5.1 Implant Shaping

There are two main strategies to implant a biomaterial in an OC defect: either placing a pre‐shaped implant within a defect or using an injectable/malleable material that can fill it. Molding is the simplest and most common method to manufacture a pre‐shaped implant (Figure 5B). In the context of OC repair, it consists in pouring/injecting a liquid precursor material in a rigid frame of the size and shape of an OC defect, and letting it harden. Interestingly, this technique allows the straightforward design of multilayer implants via a step‐by‐step molding method where layers are successively formed on top of each other.^[^ ^156^ ^]^ Using this approach, it was shown that putting layers in contact before complete hardening/gelation can improve the integration between layers (i.e., absence of discontinuity).^[^ ^161^, ^168^, ^169^ ^]^ Molding can also be used to create a bilayer scaffold with a fiber‐reinforced gel as the bottom layer by simply placing fibers in the bottom half of the mold before adding a polymer solution on top.^[^ ^170^ ^]^ Molding also allows the fabrication of oriented structures, where polymer fibers or pores are arranged along a particular axis. For example, the application of a temperature gradient from the edges to the center of a mold, which was obtained by applying liquid nitrogen to a cylindrical mold with thermally insulated bases and core, allowed to create a radially oriented structure of collagen with the aim to facilitate cell infiltration from the edges of the defect.^[^ ^171^ ^]^ This strategy enhanced in vitro BMSC infiltration and greatly improved in vivo OC regeneration in a rabbit model compared to a similar system with randomly oriented pores. More recently, a vertical temperature gradient was used to create a collagen structure that recapitulates the native vertical orientation of collagen fibers in the deep zone of cartilage.^[^ ^172^ ^]^ Overall, molding gives access to complex structures in a most simple and inexpensive way. However, from a clinical point of view, it does not allow the rapid fabrication of OC implants of any size and shape, limiting its translation. To circumvent this drawback, cutting out or punching the implant at the time of surgery has been proposed;^[^ ^147^, ^155^ ^]^ yet, the irregularities of OC defect edges make this procedure difficult. Thus, new approaches are needed, and 3D printing is attracting growing attention as a promising alternative.

Additive manufacturing, which consists of the production of 3D scaffolds in a layer‐by‐layer fashion via automated fabrication,^[^ ^173^ ^]^ was first envisioned in tissue engineering at the beginning of the 2000's as a way to fabricate personalized implants by combining medical imaging and computer‐assisted design.^[^ ^174^ ^]^ 3D printing is an additive manufacturing technique that uses printing methods (e.g., extrusion, inkjet, digital light processing) to produce 3D objects in a layer‐by‐layer fashion.^[^ ^175^ ^]^ It allows the design of implants with a high degree of structural complexity, which is of interest for OC tissue engineering. 3D printing was used to fabricate OC implants with original features, including oriented channels,^[^ ^125^ ^]^ multilayers,^[^ ^176^, ^177^ ^]^ and biomaterial gradients,^[^ ^178^ ^]^ all recapitulating part of the hierarchical organization of an OC unit. More recently, 3D bioprinting has emerged, with a set of specific 3D‐printing tools for the patterning and assembly of materials containing living elements.^[^ ^179^ ^]^ The use of the term “bioprinting” when printing only biologics as living elements is somewhat still subjected to debate. In this review, we will refer to bioprinting only when the printing of cells is involved, in accordance with a more recent definition.^[^ ^180^ ^]^ In 2016, Shim et al. were the first to implant a 3D‐bioprinted bilayer construct in vivo in rabbit OC defects. In this system, the bone layer was composed of MSCs encapsulated in a PCL fiber‐reinforced collagen gel containing BMP‐2; and the cartilage layer was composed of MSCs encapsulated in a PCL fiber‐reinforced HA hydrogel containing TGF‐*β*.^[^ ^181^ ^]^ This pioneering study led to the formation of smooth cartilage‐like tissues that were yet whiter than the surrounding healthy cartilage. Histologically, the authors observed GAG staining and Col II expression in the upper part of the newly formed tissues, while Col X was expressed in the lower part of the tissue. Subchondral bone formation was also observed on histological staining but not further assessed. More recently, 3D bioprinting was used to create a cell‐laden implant with gradients of HA and TCP concentrations, taking advantage of a homemade system for multi‐material deposition and mixing.^[^ ^99^ ^]^ In this study, IHC showed Col II and tenascin (a marker of cartilage repair) expression in the newly formed tissues, while these were absent in the untreated control group. However, the authors did not include a group treated with a continuous or bilayer implant, which does not fully allow to conclude on the benefits of a gradient structure.

As the field continues to evolve, new concepts are being explored. For example, in situ 3D printing, where a construct is printed directly in the defect/wound site during surgery, was recently reported for skin and bone regeneration,^[^ ^182^, ^183^ ^]^ as well as in the context of OC repair.^[^ ^184^ ^]^ In the latter study, OC defects were created in the trochlear groove of rabbits, and a methacrylated HA hydrogel of the same geometry was directly printed in the cavity under anesthesia. 12 weeks after surgery, the authors observed similar results with the in situ printed materials compared to the traditionally 3D‐printed and implanted materials, in terms of mechanical properties, macroscopic scores, histological aspect, and staining. Although the geometry of the reported implant was determined before surgery, and was relatively simple (i.e., a cylinder corresponding to the defect), in situ 3D printing or bioprinting could theoretically be performed based on high‐resolution scanners, for any given shape.^[^ ^185^ ^]^ It is very likely that technological progress in the field of additive manufacturing will continue, providing tools to create even more complex structures to support OC regeneration.

From a clinical point of view, pre‐formed implants have the major disadvantage to require open surgery for implantation. In particular, it is established that bleeding in the joint should be avoided as it constitutes a source of damage to articular cartilage,^[^ ^186^ ^]^ causing inflammation and possibly leading to secondary osteoarthritis.^[^ ^187^ ^]^ To address this issue, injectable materials that could be administered in a minimally‐invasive manner are of particular interest. To be injectable, a material must have a low viscosity or viscoelastic properties allowing it to flow through a needle, and be able to harden once injected. In the general context of tissue engineering, tremendous efforts have been put into the development of injectable materials with different forms (e.g., paste, cement, hydrogel) that are now being translated to the clinic.^[^ ^188^ ^]^ Yet, for OC regeneration, our work reveals that injectable biomaterials have been used only in a small portion of the reported strategies. These strategies are generally based on in situ forming materials, where liquid precursors are injected to fill an OC defect before hardening/gelation. In situ forming materials can be obtained in different ways, including: the mixing of two precursor solutions to initiate gelation right before injection;^[^ ^189^, ^190^ ^]^ the injection of a crosslinking agent on top of the injected solution;^[^ ^191^ ^]^ the chemical modification of a polymer with thermoresponsive moieties that trigger gelation at physiological temperature upon injection;^[^ ^192^ ^]^ and the exposure to UV light of a photocrosslinkable solution after injection.^[^ ^115^ ^]^ Regarding the latter strategy, it is important to mention that the in situ exposure to UV currently necessitates open surgery, nullifying the benefits of using an injectable material for minimal invasion. Nonetheless, the recent report in the cardiovascular field of the use of a microfluidic device with an integrated optical fiber for in situ photocrosslinking is opening new perspectives for arthroscopically‐crosslinkable materials.^[^ ^193^ ^]^ A major limitation of using injectable scaffolds for OC repair is the difficulty to reproduce gradients and complex structures in situ. With this in mind, a few research groups recently investigated the feasibility of delivering bilayer implants by sequential injections of two solutions, corresponding to the two main layers of an OC tissue.^[^ ^194^, ^195^, ^196^ ^]^ In particular, this approach was used to generate an injectable cellularized material with two layers (agarose that is thermosensitive in both layers, combined with alginate that is responsive to ionic concentration in the cartilage layer, and combined with BGs in the subchondral bone layer).^[^ ^100^ ^]^ The injection of multilayered solutions constitutes one possible avenue for future research.

### 5.2 Retention Methods

OC implants typically require to be physically maintained in the defect for successful tissue regeneration; and the applied retention method is therefore an important aspect of the proposed system. Yet, it is most often under‐considered in preclinical models, and the majority of the biomaterial‐assisted strategies investigated to date did not use any retention technique (Figure 5C). Various approaches exist to stabilize an OC implant, including the use of fibrin glue,^[^ ^146^, ^197^ ^]^ the use of a periosteal flap (i.e., a flap from the periosteum of the proximal tibia, sutured on top of the defect),^[^ ^11^, ^121^ ^]^ suturing,^[^ ^79^ ^]^ and press‐fitting.^[^ ^63^, ^198^ ^]^ This last method, which consists in applying pressure on the implant so that it is wedged in the defect, is frequently used in orthopedics and has therefore been used in most preclinical studies reporting a retention method. Regarding fibrin glue, its use has been widely described for the retention of cells, biomaterials, or grafts, both in pre‐clinical and clinical research.^[^ ^199^, ^200^ ^]^ However, this technique remains controversial, mainly because of its potential role in inflammation, especially for xenogeneic fibrin.^[^ ^201^ ^]^ Additionally, the supraphysiological concentrations of fibrinogen and thrombin in fibrin sealants can limit cell migration and matrix deposition, and therefore impede adequate OC regeneration.^[^ ^202^ ^]^ It is worth noting that other surgical glues exist, including collagen, albumin, polyurethane, PEG, and cyanoacrylate sealants,^[^ ^203^ ^]^ but have only been rarely investigated. In a recent study, the efficacy of several retention methods in maintaining a hydrogel scaffold (methacrylated HA gel) in a defect was studied.^[^ ^204^ ^]^ Comparing fibrin glue and press‐fit fixation, the authors showed that both techniques led to implant displacement in vivo, and thus were not adequate for such a soft hydrogel scaffold. As soft materials constitute the vast majority of the proposed cartilage‐regenerating systems, the displacement or unwanted removal of the implant could partly explain the frequent failure of these strategies.

## 6 Discussion

Despite more than two decades of effort, OC regeneration remains a major challenge to date. By scrutinizing and putting into perspective the methodologies and outcomes of almost 300 studies (reviewing method and complete table provided as Supporting Information), we drew a complete and representative overview of the field, allowing us to identify limitations and key elements for success. Our analysis highlighted the multiplicity of strategies that have been envisioned, along with the unlimited number of possible combinations of biomaterials, cell types, and bioactive molecules. More importantly, our study highlighted the variety of animal models, length of animal studies, assessment methods, and control conditions (e.g., empty defect, microfracture, commercial scaffold). Unfortunately, this renders any systematic review of the preclinical outcomes and any correlation between strategies and in vivo results nearly impossible to make. Therefore, a first step toward progressing in OC regeneration using material‐assisted tissue engineering strategies, will reside in using common and more complete evaluation methods, following clear guidelines approved by the international community. Based on our study, we believe that these should systematically include: i) subchondral bone analysis via micro‐CT and histological staining highlighting mineralized bone tissues such as Movat's pentachrome or Goldner's trichrome; ii) articular cartilage analysis via GAG staining and IHC for Col I, Col II and Col X; and iii) relevant macroscopic and histological scores, namely the ICRS macroscopic score and Sellers’ histological score. Ideally, biomechanical analyses, and lubrification evaluation when possible, should also be performed to further assess the effectiveness of regeneration. These analyses should be conducted at several time points, and include long‐term evaluation, which would allow to better evaluate subchondral bone and cartilage remodeling, as well as potential deleterious effects of the proposed treatments, such as tissue degeneration and early signs of osteoarthritis. Monitoring animal behavior, with gait analysis for instance, would also be of major value; yet, it remains surprisingly marginal.

Beyond the variability in efficacy assessment methods, several factors could explain the limited success observed to date. First, although there is a growing interest in subchondral bone regeneration, OC regeneration strategies have largely been limited to addressing the sole repair of articular cartilage, as evidenced by the rare and limited analysis of subchondral bone repair, and the predominance of single‐layer approaches. Furthermore, considering the complexity of the tissue to regenerate, most studies only focused on one particular aspect of OC regeneration (e.g., appropriate cell types, adequate combination of bioactive molecules, optimal biomaterial scaffold), possibly limiting their impact on tissue repair.

Notwithstanding the overall limited progress, systematically reviewing recent literature and putting into perspective the reported studies with a most careful second opinion on their data, allowed us to foresee some more promising avenues of research. Here is, according to us, a shortlist of approaches with the most convincing results. First, among all of the different biomaterials reported, it appears that decellularized ECM led to some of the best regeneration outcomes in different animal models, including rabbits,^[^ ^145^, ^146^, ^147^, ^205^ ^]^ but also more clinically relevant models such as beagle dogs^[^ ^129^ ^]^ and goats.^[^ ^66^ ^]^ When using synthetic materials, multilayer materials comprising BGs in the subchondral bone layer mainly showed excellent results,^[^ ^100^, ^156^, ^161^, ^176^ ^]^ which has to be tempered due to the limited number of studies to date. In general, various materials featuring multiple‐layer structures,^[^ ^62^, ^65^, ^206^ ^]^ gradients,^[^ ^177^ ^]^ temperature or ionic force‐response,^[^ ^100^ ^]^ dual‐bioactivity,^[^ ^107^, ^127^ ^]^ or fiber‐reinforcement^[^ ^61^ ^]^ have shown encouraging outcomes. In this context, the current in vitro development of more sophisticated systems, capable of mimicking both the mechanical and structural properties of a complete OC unit, seems to pave the road for improvements. For example, a trilayer hydrogel scaffold was recently designed to recapitulate the native organization of articular cartilage ECM, using microribbons of gelatin and chondroitin sulfate differently organized in space.^[^ ^207^ ^]^ This strategy led to the successful zonal differentiation of embedded human MSCs into chondrocytes secreting ECM of different compositions and mechanical properties in vitro after 21 days, as evidenced by local differences in collagen and GAG contents, and different compressive moduli between layers. It further positions 3D (bio)printing as a technology of choice for advanced OC implant design. Along with advanced biomaterial design, the controlled release of bioactive molecules to stimulate cell invasion and/or differentiation constitutes another promising approach. Bioactive molecules are increasingly used over cells, possibly owing to easier clinical translation perspectives, with overall very positive results. The use of bioactive molecules, especially GFs, almost systematically improved outcomes compared to biomaterials alone. Furthermore, when used in combination, GFs had most often synergistic effects.^[^ ^119^, ^120^, ^208^ ^]^ This could explain why PRP, which contains an ill‐defined cocktail of GFs, has been shown to significantly improve the OC healing potential.^[^ ^121^, ^122^, ^123^ ^]^ Yet, identifying key GF cocktails for efficient regeneration of the OC unit remains a challenge, which will require further fundamental research on articular tissue development and on the physiopathology of diseased OC units.

In parallel to these exciting developments, emerging technologies and recent discoveries are creating new opportunities that remain to be explored. For example, while the delivery of small molecule drugs and therapeutic proteins has been extensively studied, micro‐RNAs (miRNAs) have attracted little attention in the field. These small non‐coding RNA fragments play a key role in the regulation of gene expression,^[^ ^209^ ^]^ and have been shown to improve regeneration in other fields such as neurorestoration^[^ ^210^, ^211^ ^]^ and cardiac tissue regeneration.^[^ ^212^ ^]^ Interestingly, miRNAs are partly responsible for entire limb regeneration in other organisms such as axolotls,^[^ ^213^ ^]^ and it was recently discovered that humans possess similar miRNAs, yet in much lower quantities. MiRNAs have been associated with the turnover of articular cartilage proteins,^[^ ^214^ ^]^ the osteogenic and chondrogenic differentiation of MSCs,^[^ ^215^ ^]^ and some anti‐inflammatory mechanisms,^[^ ^216^ ^]^ demonstrating their potential as the most powerful therapeutic tools yet to be investigated for OC repair.

Immunomodulatory strategies, where inflammation is finely controlled to avoid chronicity to improve regeneration, is just another avenue to explore. Depending on the nature and the location of the target tissue, the type and duration of the immune response, and the immune cells involved, inflammation can have either a positive or negative impact.^[^ ^217^ ^]^ In cartilage, for instance, pro‐inflammatory cytokines have shown deleterious effects on chondrogenesis.^[^ ^218^ ^]^ Similarly, in bone tissues, an excess or a chronic expression of pro‐inflammatory cytokines leads to bone resorption.^[^ ^219^ ^]^ Yet, inflammation is essential to trigger bone formation and repair,^[^ ^220^ ^]^ which suggests that the use of strategies able to locally and finely tune the magnitude and duration of inflammation could be an interesting approach. In a recent article, an anti‐inflammatory molecule (honokiol) delivered in a PEG/decellularized ECM hydrogel led to more bone formation and better cartilage repair (more GAGs staining, better histological organization, increased Col II and decreased Col I expression) compared to the same hydrogel without the anti‐inflammatory molecule,^[^ ^221^ ^]^ highlighting the potential of such immunomodulation strategies. However, the implication of inflammation in regenerative processes is very complex and poorly understood, currently limiting our ability to control its underlying mechanisms.^[^ ^222^ ^]^ Nonetheless, the emerging field of immuno‐engineering that focuses on the design of biomaterials modulating the immune response, offers new perspectives for the successful restoration of tissue functions.^[^ ^223^, ^224^ ^]^ Interestingly, it was recently demonstrated that immunomodulation could be achieved through mechanical stimulation. Indeed, mechanical loading has positive effects on the proliferation and chondrogenic differentiation of MSCs through pathways involving anti‐inflammatory signals.^[^ ^33^ ^]^ This has been investigated in a few publications where rabbit OC defects were treated with PLGA implants before subjecting the animals to loading rehabilitation exercises^[^ ^123^, ^198^ ^]^ or continuous passive motion.^[^ ^225^ ^]^ In these studies, compared to sedentary animals, which exhibited only partial repair, mechanical stimulation led to an improved regeneration of both the subchondral bone and hyaline‐like cartilage, including the appearance of columnar chondrocytes and the expression of abundant GAGs and Col II. Lower levels of inflammation markers in the regenerated tissues were observed, supporting the immunomodulatory benefits of mechanical stimulation. While continuous passive motion has long shown improvement of recovery after joint trauma or surgery,^[^ ^226^ ^]^ these exciting results call for further investigations of the benefits of mechanical loading in the context of OC regeneration.

## 7 Conclusion

In this review, we reported the most recent advances in the design of material‐assisted strategies for the repair of OC defects, which constitute one of the major risk factors for osteoarthritis. Despite the tremendous amount of effort dedicated to this challenge over the past decades, the overall progress seems limited, indicating a long road to go. The careful analysis of the current state of the art has revealed the emergence of promising strategies, including injectable multilayer materials and 3D‐(bio)printed biomimetic implants. Along with advanced biotechnologies, the booming development of innovative biomaterials will certainly soon be a game‐changer in the way the problem is approached. Particular attention should be paid to the design of biomaterial scaffolds allowing the sequential delivery of adequate combinations of bioactive molecules to favor progenitor cell invasion and differentiation. It will allow evolving toward cell‐free strategies, more easily meeting the stringent quality and regulatory requirements for clinical translation. By then, the community will have to find new consensus for the scoring systems, assessment methods, and animal models, allowing to build better common knowledge to tackle this challenge.

## Conflict of Interest

The authors declare no conflict of interest.

## Supporting information

Supporting Information
