# Supplementary material for: Material‐Assisted Strategies for Osteochondral Defect Repair
Source: Adv Sci (Weinh). 2022 Mar 24;9(16):2200050. doi: 10.1002/advs.202200050 (PMC9165504; doi:10.1002/advs.202200050)
Supplement: Supplementary file 1 — Supporting Information [file ADVS-9-2200050-s001.pdf]

Supplementary Information 1

1. Keyword search:

A literature search was performed in the PubMed and Web of Science databases, using the keywords “osteochondral”, “repair”, “regeneration”, “biomaterial”, with AND/OR operators. The original articles published on the subject between January 1, 2015 and November 30, 2021 were identified. After removing the duplicates, the remaining articles were screened by title and abstract. When the title and abstract were not sufficiently informative, the Material and Methods section was consulted.

2. Inclusion/exclusion criteria

Included articles were written in English, and reported the preclinical evaluation of biomaterial- assisted strategies for the repair of OC defects. Exclusion criteria were: (i) the use of a biomaterial as an intra-articular injection only (not in a defect), (ii) the evaluation of the OC regeneration potential by a subcutaneous injection only, (iii) the involvement of an osteoarthritic animal model. A total of 289 publications in accordance with our criteria were identified and used to perform a meta-analysis of the biomaterial design, the biological elements added, and the methods used for repair and/or regeneration assessment of osteochondral defects.

Supplementary Table 1 : Summary of the main strategies used

General approach			Composition of OC implants					Implantation aspects			Analysis techniques					Original articles
**Overall strategy**	**Number of layers**	**Difference between layers**	**Layer 1**	**Layer 2**	**Layer 3**	**Cell type**	**Biologics**	**Retention means**	**Implant shaping**	**Animal model**	**Histo.**	**Macro. obs.**	**IHC**	**Micro-CT**	**Biomechanics**	**References**
Biomat only	Monolayer	/	PLGA	/	/	/	/	Press-fit	Molding	Rabbit	X	X	X	X		Chang, Nai-Jen et al. “Positive effects of cell-free porous PLGA implants and early loading exercise on hyaline cartilage regeneration in rabbits.” Acta biomaterialia vol. 28 (2015): 128-137.
Biomat only	Monolayer	/	PLGA	/	/	/	/	Press-fit	Molding	Rabbit	X	X	X	X		Wang, Hsueh-Chun et al. “Intra-articular injection of N-acetylglucosamine and hyaluronic acid combined with PLGA scaffolds for osteochondral repair in rabbits.” PloS one vol. 13,12 e0209747. 31 Dec. 2018,
Biomat only	Monolayer	/	PLGA	/	/	/	/	Press-fit	Molding	Minipig	X	X	X	X		Lin, Chih-Chan et al. “Beneficial Therapeutic Approach of Acellular PLGA Implants Coupled With Rehabilitation Exercise for Osteochondral Repair: A Proof of Concept Study in a Minipig Model.” The American journal of sports medicine vol. 48,11 (2020): 2796-2807.
Biomat only	Monolayer	/	HA	/	/	/	/	/	Commercial	Rabbit	X	X				Uçan, Vahdet et al. "Comparison of Small-diameter-hole and Traditional Microfracture in Cartilage Repair and the Effect of Adding a Hyaluronic Acid-based Acellular Matrix Scaffold : An Animal Study." Bezmialem Science, 9(1), (2021) 84-90.
Biomat only	Monolayer	/	Cartilage ECM + peptide nanofiber hydrogel	/	/	/	/	/	Molding	Rabbit	X	X	X			Sun, Xun et al. “In Situ Articular Cartilage Regeneration through Endogenous Reparative Cell Homing Using a Functional Bone Marrow-Specific Scaffolding System.” ACS applied materials & interfaces vol. 10,45 (2018): 38715-38728.
Biomat only	Trilayer	Biomat	Cartilage ECM	PLGA + b-TCP	PLGA + b-TCP	/	/	Press-fit	Molding + 3D-printing	Goat	X	X	X		X	Jia, Shuaijun et al. “Multilayered Scaffold with a Compact Interfacial Layer Enhances Osteochondral Defect Repair.” *ACS applied materials & interfaces* vol. 10,24 (2018): 20296-20305
Biomat only	Monolayer		CS + silk fibroin	/	/	/	/	/	Molding	Rabbit	X	X				Zhou, Feifei et al. “Silk fibroin-chondroitin sulfate scaffold with immuno-inhibition property for articular cartilage repair.” Acta biomaterialia vol. 63 (2017): 64-75.
Biomat only	Trilayer	Biomat	Col I + Col II + HA	Col I + Col II + HAP	Col I + HAP	/	/	Press-fit	Cut	Rabbit	X	X	X	X		Levingstone, Tanya J et al. “Multi-layered collagen-based scaffolds for osteochondral defect repair in rabbits.” Acta biomaterialia vol. 32 (2016): 149-160.
Biomat only	Bilayer	Biomat	Cartilage ECM	/	Growth plate ECMs	/	/	Press-fit	Molding	Goat	X	X	X			Cunniffe, Gráinne M et al. “Tissue-specific extracellular matrix scaffolds for the regeneration of spatially complex musculoskeletal tissues.” Biomaterials vol. 188 (2019): 63-73.
Biomat only	Monolayer	Biomat	PCL + HAP	/	/	/	/	/	3D-printing	Rabbit	X	X		X		Du, Yingying et al. “Selective laser sintering scaffold with hierarchical architecture and gradient composition for osteochondral repair in rabbits.” Biomaterials vol. 137 (2017): 37-48
Biomat only	Trilayer	Biomat	Col I + Col II + HA	Col I + HA	Col I + HAP	/	/	/	Cut	Goat	X	X		X		Levingstone, Tanya J et al. “Cell-free multi-layered collagen-based scaffolds demonstrate layer specific regeneration of functional osteochondral tissue in caprine joints.” Biomaterials vol. 87 (2016): 69-81.
Biomat only	Bilayer	Biomat	CS-MA + PNIPAAm	/	PCL + PEG	/	/	/	Other	Rabbit	X	X		X	X	Liao, Jinfeng et al. “The fabrication of biomimetic biphasic CAN-PAC hydrogel with a seamless interfacial layer applied in osteochondral defect repair.” Bone research vol. 5 17018. 4 Jul. 2017,
Biomat only	Monolayer	/	Biopolymers	/	/	/	/	/	Undefined	Rabbit	X					Albuquerque, Paulo Cezar Vidal Carneiro de et al. “A comparative study of the areas of osteochondral defects produced in femoral condyles of rabbits treated with sugar cane biopolymer gel.” Acta cirurgica brasileira vol. 30,11 (2015): 770-7.
Biomat only	Monolayer	/	CS + PEG	/	/	/	/	/	Injection	Rat	X		X			Schaeffer, Christine et al. “Injectable Microannealed Porous Scaffold for Articular Cartilage Regeneration.” Annals of plastic surgery vol. 84,6S Suppl 5 (2020): S446-S450.
Biomat only	Monolayer	/	Parylene	/	/	/	/	/	Other	Rabbit	X	X	X			Franciozi, Carlos Eduardo da Silveira et al. “Parylene scaffold for cartilage lesion.” Biomedical microdevices vol. 19,2 (2017): 26.
Biomat only	Monolayer	/	PAMPS + PDMAAm	/	/	/	/	Press-fit	Molding	Rabbit	X	X	X			Higa, Kotaro et al. “Effects of osteochondral defect size on cartilage regeneration using a double-network hydrogel.” BMC musculoskeletal disorders vol. 18,1 210. 22 May. 2017,
Biomat only	Bilayer	Biomat	PCL	/	PCL	/	/	Press-fit	3D-printing + Electrospinning + Cut	Sheep	X	X			X	Schagemann, Jan C et al. “Bilayer Implants: Electromechanical Assessment of Regenerated Articular Cartilage in a Sheep Model.” *Cartilage* vol. 7,4 (2016): 346-60.
Biomat only	Monolayer		Alginate + HAP + PLA	/		/	/	Press-fit	Other	Rabbit	X	X				Żylińska, Beata et al. “Osteochondral Repair Using Porous Three-dimensional Nanocomposite Scaffolds in a Rabbit Model.” *In vivo (Athens, Greece)* vol. 31,5 (2017): 895-903.
Biomat only	Bilayer	Biomat	Chitosan + Col	/	Chitosan + Col + HAP	/	/	/	Other	Rabbit, sheep	X	X	X	X		Roffi, Alice et al. “A Composite Chitosan-Reinforced Scaffold Fails to Provide Osteochondral Regeneration.” *International journal of molecular sciences* vol. 20,9 2227. 7 May. 2019,
Biomat only	Bilayer	Biomat	Col II + HA + PG	/	Col II + HA + HAP + PG	/	/	/	Other	Rat	X	X				Kumai, Takanori et al. “A novel, self-assembled artificial cartilage-hydroxyapatite conjugate for combined articular cartilage and subchondral bone repair: histopathological analysis of cartilage tissue engineering in rat knee joints.” *International journal of nanomedicine* vol. 14 1283-1298. 19 Feb. 2019,
Biomat only	Monolayer	/		/	HAP + PCL	/	/	/	3D-printing	Rabbit	X		X			Wei, Bo et al. “Three-dimensional polycaprolactone-hydroxyapatite scaffolds combined with bone marrow cells for cartilage tissue engineering.” *Journal of biomaterials applications* vol. 30,2 (2015): 160-70.
Biomat only	Monolayer	/	PAMPS + PDMAAm	/	/	/	/	/	Molding	Sheep	X	X				Kitamura, Nobuto et al. “In vivo cartilage regeneration induced by a double-network hydrogel: Evaluation of a novel therapeutic strategy for femoral articular cartilage defects in a sheep model.” *Journal of biomedical materials research. Part A* vol. 104,9 (2016): 2159-65.
Biomat only	Monolayer	/	HA + PNIPAAm	/	/	/	/	/	Injection	Rabbit	X	X				D'Este, Matteo et al. “Evaluation of an injectable thermoresponsive hyaluronan hydrogel in a rabbit osteochondral defect model.” Journal of biomedical materials research. Part A vol. 104,6 (2016): 1469-78.
Biomat only	Monolayer	/	Osteochondral ECM	/	/	/	/	/	Other	Rabbit	X	X		X		Hiemer, Bettina et al. “Repair of cartilage defects with devitalized osteochondral tissue: A pilot animal study.” Journal of biomedical materials research. Part B, Applied biomaterials vol. 107,7 (2019): 2354-2364.
Biomat only	Monolayer	/	AH	/	/	/	/	Hyaluronic acid gel	Punch	Rabbit	X	X				Bauer, Christoph et al. “Hyaluronan thiomer gel/matrix mediated healing of articular cartilage defects in New Zealand White rabbits-a pilot study.” Journal of experimental orthopaedics vol. 4,1 (2017): 14.
Biomat only	Bilayer	Biomat	Alginate + HA	/	Alginate + HAP	/	/	Press-fit for Sheep for Rabbit	Punch	Rabbit, sheep	X	X	X	X		Filardo, Giuseppe et al. “Novel alginate biphasic scaffold for osteochondral regeneration: an in vivo evaluation in rabbit and sheep models.” *Journal of materials science. Materials in medicine* vol. 29,6 74. 26 May. 2018,
Biomat only	Monolayer	/	Chitosan + D-(+) raffinose	/	/	/	/	Flap	Undefined	Rabbit	X	X				Ravanetti, Francesca et al. “Chitosan-based scaffold modified with D-(+) raffinose for cartilage repair: an in vivo study.” *Journal of negative results in biomedicine* vol. 14 2. 14 Jan. 2015,
Biomat only	Trilayer	Biomat	PGA	PLA	b-TCP+ Col I + PCL + PLA	/	/	/	Molding	Sheep	X	X	X			Yucekul, Altug et al. “Tri-layered composite plug for the repair of osteochondral defects: in vivo study in sheep.” *Journal of tissue engineering* vol. 8 2041731417697500. 13 Apr. 2017,
Biomat only	Monolayer	/	GelMA + HA-MA	/	/	/	/	/	Malleable	Rabbit	X	X		X		Lin, Hang et al. “Optimization of photocrosslinked gelatin/hyaluronic acid hybrid scaffold for the repair of cartilage defect.” Journal of tissue engineering and regenerative medicine vol. 13,8 (2019): 1418-1429.
Biomat only	Bilayer	/	Cartilage ECM + CaP	/	b-TCP	/	/	Press-fit	3D-printing + Molding	Horse	X	X	X	X	X	Vindas Bolaños, R A et al. “The use of a cartilage decellularized matrix scaffold for the repair of osteochondral defects: the importance of long-term studies in a large animal model.” *Osteoarthritis and cartilage* vol. 25,3 (2017): 413-420.
Biomat only	Monolayer	/	Alginate	/	/	/	/	/	Undefined	Dog	X	X	X			Onodera, Tomohiro et al. “Therapeutic effects and adaptive limits of an acellular technique by ultrapurified alginate (UPAL) gel implantation in canine osteochondral defect models.” *Regenerative therapy* vol. 14 154-159. 20 Feb. 2020,

Biomat only	Monolayer	/	BG + Fibrin	/	/	/	/	Fibrin glue	Malleable	Rabbit	X	X				Zazgyva, AncuŢa Marilena et al. "S53P4 bioactive glass and fibrin glue for the treatment of osteochondral lesions of the knee - a preliminary in vivo study in rabbits." *Romanian journal of morphology and embryology = Revue roumaine de morphologie et embryologie* vol. 56,3 (2015): 1085-90.
Biomat only	Monolayer	/	HA + heparan sulfate	/	/	/	/	/	Undefined	Rabbit	X	X	X			Lee, Jonathan H et al. "A Heparan Sulfate Device for the Regeneration of Osteochondral Defects." *Tissue engineering. Part A* vol. 25,5-6 (2019): 352-363
Biomat only	Monolayer	/	Osteochondral ECM	/	/	/	/	Press-fit	Other	Sheep	X				X	Novak, Tyler et al. "In Vivo Cellular Infiltration and Remodeling in a Decellularized Ovine Osteochondral Allograft." *Tissue engineering. Part A* vol. 22,21-22 (2016): 1274-1285.
Biomat only	Monolayer	/	PLGA + amnion-derived ECM	/	/	/	/	/	Cut	Rat	X		X			Nogami, Makiko et al. "A Human Amnion-Derived Extracellular Matrix-Coated Cell-Free Scaffold for Cartilage Repair: In Vitro and In Vivo Studies." Tissue engineering. Part A vol. 22,7-8 (2016): 680-8.
Biomat only	Monolayer	/	PCL	/	/	/	/	Fibrin glue	3D-printing (study 1)<br>3D-printing + Molding (study 3)	Horse	X			X		Mancini, Irina A D et al. "Fixation of Hydrogel Constructs for Cartilage Repair in the Equine Model: A Challenging Issue." Tissue engineering. Part C, Methods vol. 23,11 (2017): 804-814.
Biomat only	Monolayer	/	Alginate	/	/	/	/	/	Injection	Rabbit	X	X		X		Baba, Rikiya et al. "A Novel Bone Marrow Stimulation Technique Augmented by Administration of Ultrapurified Alginate Gel Enhances Osteochondral Repair in a Rabbit Model." *Tissue engineering. Part C, Methods* vol. 21,12 (2015): 1263-73.
Biomat only	Bilayer	Biomat	PU	/	PEEK	/	/	Press-fit	Undefined	Poney	X		X			Korthagen, N M et al. "A short-term evaluation of a thermoplastic polyurethane implant for osteochondral defect repair in an equine model." *Veterinary journal (London, England : 1997)* vol. 251 (2019): 105340.
Biomat only	Bilayer	Biomat	Cartilage ECM	/	Osteochondral ECM	/	/	/	Undefined	Rabbit	X		X			Lin, X et al. "Biphasic hierarchical extracellular matrix scaffold for osteochondral defect regeneration." *Osteoarthritis and cartilage* vol. 26,3 (2018): 433-444.
Biomat only	Bilayer	Biomat	Silk fibroin	/	Silk fibroin + CaP	/	/	Press-fit	Molding	Rabbit	X	X	X	X		Yan, Le-Ping et al. "Bilayered silk/silk-nanoCaP scaffolds for osteochondral tissue engineering: In vitro and in vivo assessment of biological performance." *Acta biomaterialia* vol. 12 (2015): 227-241.
Biomat only	Monolayer	/	PLGA + magnesium hydroxide	/	/	/	/	/	Molding	Rat	X	X	X			Park, Kwang-Sook et al. "Versatile effects of magnesium hydroxide nanoparticles in PLGA scaffold-mediated chondrogenesis." *Acta biomaterialia* vol. 73 (2018): 204-216.
Biomat only	Bilayer	Biomat	Col	/	BCP	/	/	/	Molding + other	Rabbit	X	X	X	X		Seong, Yun-Jeong et al. "Calcium Phosphate-Collagen Scaffold with Aligned Pore Channels for Enhanced Osteochondral Regeneration." Advanced healthcare materials vol. 6,24 (2017): 10.1002/adhm.201700966.
Biomat only	Monolayer	/	HAP + PAMPS + PDMAAm	/	/	/	/	/	Molding	Rabbit	X			X		Nonoyama, Takayuki et al. "Double-Network Hydrogels Strongly Bondable to Bones by Spontaneous Osteogenesis Penetration." Advanced materials (Deerfield Beach, Fla.) vol. 28,31 (2016): 6740-5.
Biomat only	Bilayer	Biomat	PACG + GelMA	/	BG + GelMA + PACG	/	/	/	3D-printing	Rat	X		X	X		Gao, Fei et al. "Osteochondral Regeneration with 3D-Printed Biodegradable High-Strength Supramolecular Polymer Reinforced-Gelatin Hydrogel Scaffolds." *Advanced science (Weinheim, Baden-Wurttemberg, Germany)* vol. 6,15 1900867. 11 Jun. 2019,
Biomat only	Bilayer	Biomat	Aragonite	/	Aragonite	/	/	Press-fit	Commercial	Goat	X	X	X	X		Kon, Elizaveta et al. "Reconstruction of Large Osteochondral Defects Using a Hemicondylar Aragonite-Based Implant in a Caprine Model." *Arthroscopy : the journal of arthroscopic & related surgery : official publication of the Arthroscopy Association of North America and the International Arthroscopy Association* vol. 36,7 (2020): 1884-1894.
Biomat only	Monolayer	/	Glycopeptide GAG-like	/	/	/	/	/	Malleable	Rabbit	X		X			Ustun Yaylaci, Seher et al. "Supramolecular GAG-like Self-Assembled Glycopeptide Nanofibers Induce Chondrogenesis and Cartilage Regeneration." *Biomacromolecules* vol. 17,2 (2016): 679-89.
Biomat only	Monolayer	/		/	Bone ECM + Col or PEG + HPMC	/	/	/	Malleable	Rabbit	X		X			Giannoni, Paolo et al. "Rheological properties, biocompatibility and in vivo performance of new hydrogel-based bone fillers." *Biomaterials science* vol. 4,11 (2016): 1691-1703.
Biomat only	Bilayer	Biomat	PEG	/	BG	/	/	/	Molding	Rabbit	X		X	X		Lin, Dan et al. "A viscoelastic PEGylated poly(glycerol sebacate)-based bilayer scaffold for cartilage regeneration in full-thickness osteochondral defect." *Biomaterials* vol. 253 (2020): 120095.
Biomat only	Bilayer	Biomat	Alginate + CS + PVA	/	Alginate + HAP + PVA	/	/	/	Injection	Rabbit	X	X		X		Radhakrishnan, Janani et al. "Gradient nano-engineered in situ forming composite hydrogel for osteochondral regeneration." *Biomaterials* vol. 162 (2018): 82-98.
Biomat only	Monolayer	/	Si + CaP	/	/	/	/	/	Other	Rabbit	X	X		X		Bunpetch, Varitsara et al. "Silicate-based bioceramic scaffolds for dual-lineage regeneration of osteochondral defect." *Biomaterials* vol. 192 (2019): 323-333.
Biomat only	Monolayer	/	PCL + PSPU-U	/		/	/	Press-fit	Commercial	Rat	X	X	X			Shah, Sarav S et al. "Optimization of Degradation Profile for New Scaffold in Cartilage Repair." *Cartilage* vol. 9,4 (2018): 438-449.
Biomat only	Monolayer	/	AH-MA + PEG-MA	/	/	/	/	/	Molding vs 3D-printing	Rabbit	X	X	X			Ma, Kaiwei et al. "Application of robotic-assisted *in situ* 3D printing in cartilage regeneration with HAMA hydrogel: An *in vivo* study." *Journal of advanced research* vol. 23 123-132. 28 Jan. 2020,
Biomat only	Bilayer	Biomat	Col I	/	Col I + HAP-Mg or wollastonite or wollastonite + HAP	/	/	Press-fit	Molding	Sheep	X	X		X		Gervaso, F et al. "Comparison of three novel biphasic scaffolds for one-stage treatment of osteochondral defects in a sheep model." Journal of biological regulators and homeostatic agents vol. 30,4 Suppl 1 (2016): 24-31.
Biomat only	Bilayer	Biomat	Silk fibroin	/	Silk fibroin	/	/	Press-fit	Molding	Rabbit	X	X		X		Singh, Yogendra Pratap et al. "Hierarchically structured seamless silk scaffolds for osteochondral interface tissue engineering." Journal of materials chemistry. B vol. 6,36 (2018): 5671-5688.
Biomat only	Monolayer	/	Col + Silk fibroin	/		/	/	/	Molding	Rabbit	X		X	X		Feng, Xue et al. "Influence of pore architectures of silk fibroin/collagen composite scaffolds on the regeneration of osteochondral defects in vivo." Journal of materials chemistry. B vol. 8,3 (2020): 391-405.
Biomat only	Bilayer	Biomat	Osteochondral grafts of demineralized bone ECM with protected cartilage	/	Bone ECM	/	/	Press-fit	Other	Rabbit	?	?	?	?	?	Li, Siming et al. "Repair of massively defected hemi-joints using demineralized osteoarticular allografts with protected cartilage." Journal of materials science. Materials in medicine vol. 26,8 (2015): 227.
Biomat only	Monolayer	/	Gelatin or polyglycerol sebacate	/	/	/	/	Blood clot	Undefined	Goat	X		X			Chin, Adam R et al. "Regenerative Potential of Various Soft Polymeric Scaffolds in the Temporomandibular Joint Condyle." Journal of oral and maxillofacial surgery : official journal of the American Association of Oral and Maxillofacial Surgeons vol. 76,9 (2018): 2019-2026.
Biomat only	Bilayer	Biomat	HA	/	Aragonite	/	/	/	Undefined	Goat	X	X	X	X		Kon, Elizaveta et al. "Osteochondral regeneration with a novel aragonite-hyaluronate biphasic scaffold: up to 12-month follow-up study in a goat model." Journal of orthopaedic surgery and research vol. 10 81. 28 May. 2015,
Biomat only	Monolayer	/	BG + PLGA	/	/	/	/	Press-fit	Cut	Rabbit	X	X		X		Salonius, Eve et al. "Gas-foamed poly(lactide-co-glycolide) and poly(lactide-co-glycolide) with bioactive glass fibres demonstrate insufficient bone repair in lapine osteochondral defects." Journal of tissue engineering and regenerative medicine vol. 13,3 (2019): 406-415.
Biomat only	Monolayer	/	PLGA	/	/	/	/	/	Molding	Rabbit	X	X	X			Dai, Yuankun et al. "Regeneration of osteochondral defects in vivo by a cell-free cylindrical poly(lactide-co-glycolide) scaffold with a radially oriented microstructure." Journal of tissue engineering and regenerative medicine vol. 12,3 (2018): e1647-e1661.
Biomat only	Monolayer	/	GelMA + HA-MA	/	/	/	/	/	Molding	Rabbit	X	X		X		Lin, Hang et al. "Optimization of photocrosslinked gelatin/hyaluronic acid hybrid scaffold for the repair of cartilage defect." Journal of tissue engineering and regenerative medicine vol. 13,8 (2019): 1418-1429.
Biomat only	Monolayer	/	Cartilage or osteochondral ECM	/	/	/	/	/	Cut	Rabbit	X	X	X			Lin, X et al. "Biphasic hierarchical extracellular matrix scaffold for osteochondral defect regeneration." Osteoarthritis and cartilage vol. 26,3 (2018): 433-444.
Biomat only	Monolayer	/	BG + Pluronic F-127	/	/	/	/	/	3D-printing	Rabbit	X	X		X		Dang, Wentao et al. "3D printing of Mo-containing scaffolds with activated anabolic responses and bi-lineage bioactivities." Theranostics vol. 8,16 4372-4392. 30 Jul. 2018,
Biomat only	Monolayer	/	Alginate + BG + Pluronic F-128	/	/	/	/	/	3D-printing	Rabbit	X	X		X		Deng, Cuijun et al. "Bioactive Scaffolds for Regeneration of Cartilage and Subchondral Bone Interface." *Theranostics* vol. 8,7 1940-1955. 15 Feb. 2018,
Biomat only	Monolayer	/	BG-ceramics	/	/	/	/	/	3D-printing	Rabbit	X	X		X		Lin, Rongcai et al. "Copper-incorporated bioactive glass-ceramics inducing anti-inflammatory phenotype and regeneration of cartilage/bone interface." Theranostics vol. 9,21 6300-6313. 14 Aug. 2019,
Biomat only	Bilayer + Monolayer	Biomat	Col I	/	Col I + HAP-Mg or wollastonite or wollastonite + HAP	/	/	Press-fit	Molding	Sheep		X				Crovace, Alberto M et al. "Evaluation of in Vivo Response of Three Biphasic Scaffolds for Osteochondral Tissue Regeneration in a Sheep Model." Veterinary sciences vol. 6,4 90. 9 Nov. 2019,
Biomat only	Bilayer	Biomat	Chitosan-Si + HPMC-Si	/	BCP	/	/	/	Injection	Sheep	X	X	X			live, Mélanie et al. "Preliminary evaluation of an osteochondral autograft, a prosthetic implant, and a biphasic absorbable implant for osteochondral reconstruction in a sheep model." Veterinary surgery : VS vol. 49,3 (2020): 570-581.
Biomat only	Monolayer	/	Alginate + bioceramic + Pluronic F-127	/	/	/	/	/	3D-printing	Rabbit	X	X		X		Chen, Lei et al. "3D printing of a lithium-calcium-silicate crystal bioscaffold with dual bioactivities for osteochondral interface reconstruction." Biomaterials vol. 196 (2019): 138-150.
Biomat only	Monolayer	/	Alginate + bioceramic + Pluronic F-127	/	/	/	/	/	3D-printing	Rabbit	X	X		X		Deng, Cuijun et al. "Bioactive Scaffolds for Regeneration of Cartilage and Subchondral Bone Interface." Theranostics vol. 8,7 1940-1955. 15 Feb. 2018,
Biomat only	Monolayer	/	Peptide-based hydrogels + PCL	/	/	/	/	/	3D-printing	Rabbit	X	X		X		Li, Lan et al. "3D Molecularly Functionalized Cell-Free Biomimetic Scaffolds for Osteochondral Regeneration." Advanced Functional Materials, 29(6), (2018) 1807356.

Biomat only	Trilayer	Biomat	Col I	Col I + HAP-Mg	Col I + HAP-Mg	/	/	/	Undefined	Rat	X	X				Solak, Kazim et al. "Histological Comparison of Nanocomposite Multilayer Biomimetic Scaffold, A Chondral Scaffold, and Microfracture Technique to Repair Experimental Osteochondral Defects in Rats." *The Eurasian journal of medicine* vol. 52,2 (2020): 145-152.
Biomat only	Bilayer	Biomat	Col + HA	/	Col 10 + HA + HAP	/	/	/	Molding	Rabbit	X	X	X	X		Liu, KaiQiang et al. "A biomimetic bi-layered tissue engineering scaffolds for osteochondral defects repair." Sci. China Technol. Sci. 64, 793–805 (2021)
Biomat only	Monolayer	/	Chitosan + Col + Fibrin + + PCL + PEG	/	/	/	/	Tisseel Lyo glue	Combination	Rabbit	X		X			Filová, Eva et al. "Hydrogel Containing Anti-CD44-Labeled Microparticles, Guide Bone Tissue Formation in Osteochondral Defects in Rabbits." *Nanomaterials (Basel, Switzerland)*vol. 10,8 1504. 31 Jul. 2020,
Biomat only	Bilayer	Biomat	Gellan gum	/	Demineralized bone particles+ gellan gum	/	/	/	Molding	Rabbit	X		X			Choi, Joo Hee et al. "Characterization and Potential of a Bilayered Hydrogel of Gellan Gum and Demineralized Bone Particles for Osteochondral Tissue Engineering." ACS applied materials & interfaces vol. 12,31 (2020): 34703-34715.
Biomat only	Monolayer	/	Peptide-based hydrogels	/	/	/	/	/	Injection	Rabbit	X	X	X			Lv, Xiao et al. "Simultaneous Recruitment of Stem Cells and Chondrocytes Induced by a Functionalized Self-Assembling Peptide Hydrogel Improves Endogenous Cartilage Regeneration." *Frontiers in cell and developmental biology* vol. 8 864. 27 Aug. 2020,
Biomat only	Bilayer	Biomat	Col + CS	/	Col + HAP	/	/	/	Molding	Rabbit	X	X	X	X		Zhou, Haichao, et al. "Biphasic fish collagen scaffold for osteochondral regeneration." Materials & Design, 195, (2020) 108947. https://doi.org/10.1016/j.matdes.2020.108947
Biomat only	Monolayer	/	/	/	PLGA + Col I<br>or<br>PLGA + HAP	/	/	/	Molding	Rabbit	X	X		X		Krok-Borkowicz, Małgorzata et al. "Surface-Modified Poly(l-lactide-*co*-glycolide) Scaffolds for the Treatment of Osteochondral Critical Size Defects-In Vivo Studies on Rabbits." *International journal of molecular sciences* vol. 21,20 7541. 13 Oct. 2020,
Biomat only	Bilayer	Biomat	GelMa	GelMA + HAP	/	/	/	/	Malleable	Rabbit	X	X	X	X		Gao, Jingming et al. "Cell-Free Bilayered Porous Scaffolds for Osteochondral Regeneration Fabricated by Continuous 3D-Printing Using Nascent Physical Hydrogel as Ink." *Advanced healthcare materials* vol. 10,3 (2021): e2001404.
Biomat only	Monolayer	/	ECM	/	/	/	/	/	Malleable	Rabbit	X	X	X	X		Wang, Zhifa et al. "Extracellular matrix derived from allogenic decellularized bone marrow mesenchymal stem cell sheets for the reconstruction of osteochondral defects in rabbits." *Acta biomaterialia* vol. 118 (2020): 54-68.
Biomat only	Monolayer	/	Silk fibroin	/	/	/	/	/	Cut	Rabbit	X	X	X	X		Zhang, Wei et al. "Cell-Free Biomimetic Scaffold with Cartilage Extracellular Matrix-Like Architectures for *In Situ* Inductive Regeneration of Osteochondral Defects." *ACS biomaterials science & engineering* vol. 6,12 (2020): 6917-6925.
Biomat only	Bilayer	Biomat	CS + silk fibroin	/	CS + silk fibroin + HAP	/	/	/	Molding	Rat	X	X	X	X		Shang, Lingling et al. "Nanotextured silk fibroin/hydroxyapatite biomimetic bilayer tough structure regulated osteogenic/chondrogenic differentiation of mesenchymal stem cells for osteochondral repair." *Cell proliferation* vol. 53,11 (2020): e12917.
Biomat only	Bilayer	Biomat	Chitosan	/	b-TCP + chitosan	/	/	/	Cut	Rat	X	X	X	X		Xu, Dongdong et al. "Bi-layered Composite Scaffold for Repair of the Osteochondral Defects." *Advances in wound care* vol. 10,8 (2021): 401-414.
Biomat only	Monolayer	/	Gelatin + GelMA + oxidized dextran	/	/	/	/	/	Injection	Rabbit	X	X	X		X	Zhou, Feifei et al. "Tough hydrogel with enhanced tissue integration and in situ forming capability for osteochondral defect repair." Applied Materials Today, 13, (2018) 32-44.
Biomat only	Bilayer	Biomat	Cartilage ECM	/	Bone ECM	/	/	/	Molding	Rabbit	X	X	X	X		Cao, Runfeng et al. "A Biomimetic Biphasic Scaffold Consisting of Decellularized Cartilage and Decalcified Bone Matrixes for Osteochondral Defect Repair." *Frontiers in cell and developmental biology* vol. 9 639006. 19 Feb. 2021,
Biomat only	Monolayer	/	Silfk fibroin + peptide elastin-like	/	/	/	/	/	Undefined	Rabbit	X	X	X	X	X	Chen, Zhuoyue et al. "Elastin-like polypeptide modified silk fibroin porous scaffold promotes osteochondral repair." *Bioactive materials* vol. 6,3 589-601. 18 Sep. 2020,
Biomat only	Monolayer	/	Chitosan + PHB	/	/	/	/	/	Undefined	Goat	X	X		X		Petrovova, Eva et al. "PHB/CHIT Scaffold as a Promising Biopolymer in the Treatment of Osteochondral Defects-An Experimental Animal Study." *Polymers* vol. 13,8 1232. 11 Apr. 2021,
Biomat only	Monolayer	/	Cartilage ECM particles + Chitosan + PLGA	/	/	/	/	/	Molding	Rabbit	X	X	X			Shen, Yanbing et al. "Engineering a Highly Biomimetic Chitosan-Based Cartilage Scaffold by Using Short Fibers and a Cartilage-Decellularized Matrix." *Biomacromolecules* vol. 22,5 (2021): 2284-2297.
Biomat only	Monolayer	/	HA	/	/	/	/	/	Injection	Rat	X	X	X			Chen, Jiaqing et al. "Modified hyaluronic acid hydrogels with chemical groups that facilitate adhesion to host tissues enhance cartilage regeneration." *Bioactive materials* vol. 6,6 1689-1698. 29 Nov. 2020,
Biomat only	Bilayer	Biomat	Col II + CS + PVA	/	BCP + carbon nanotube + PVA	/	/	/	Molding	Rabbit	X	X	X	X	X	Lan, Weiwei et al. "Physicochemical properties and biocompatibility of the bi-layer polyvinyl alcohol-based hydrogel for osteochondral tissue engineering." Materials & Design, 204, (2021) 109652.
Biomat only	Monolayer	/	Silk-naNosilicate	/	/	/	/	/	Undefined	Rabbit	X	X	X	X		Zhang, Wei et al. "Enzymatically crosslinked silk-nanosilicate reinforced hydrogel with dual-lineage bioactivity for osteochondral tissue engineering." *Materials science & engineering. C, Materials for biological applications* vol. 127 (2021): 112215.
Biomat only	Trilayer	Biomat	CS + PLCL	PLCL	BG + PLCL	/	/	/	Undefined	Rabbit	X	X		X		Liu, Mengtao et al. "Artificial osteochondral interface of bioactive fibrous membranes mediating calcified cartilage reconstruction." *Journal of materials chemistry. B* vol. 9,37 7782-7792. 29 Sep. 2021,
Biomat only	Monolayer	/	PVA	/	/	/	/	/	Commercial	Sheep	X	X	X	X		Cercone, Marta et al. "Early Failure of a Polyvinyl Alcohol Hydrogel Implant With Osteolysis and Foreign Body Reactions in an Ovine Model of Cartilage Repair." *The American journal of sports medicine* vol. 49,12 (2021): 3395-3403.
Biomat only	Bilayer	Biomat	Col + PLGA	Titanium alloy	/	/	/	/	3D-printing	Rabbit	X	X		X	X	Yang, Tao et al. "Bilayered scaffold with 3D printed stiff subchondral bony compartment to provide constant mechanical support for long-term cartilage regeneration." *Journal of orthopaedic translation* vol. 30 112-121. 12 Oct. 2021,
Biomat only	Monolayer	/	Cartilage ECM	/	/	/	/	/	Undefined	Rabbit	X	X	X			Chu, Wenhui et al. "The use of a novel deer antler decellularized cartilage-derived matrix scaffold for repair of osteochondral defects." *Journal of biological engineering* vol. 15,1 23. 3 Sep. 2021,
Biomat only	Bilayer	Biomat	Acryloyl glucosamine + GelMA	/	Vinylphosphonic acid	/	/	/	Molding	Rabbit	X	X	X	X		Chen, Zhuoxin et al. "Heterogenous hydrogel mimicking the osteochondral ECM applied to tissue regeneration." *Journal of materials chemistry. B* vol. 9,41 8646-8658. 27 Oct. 2021,
Biomat only	Bilayer	Biomat	Gellan Gum+alginate	/	Gellan gum + HAP	/	/	/	Molding	Rabbit	X	X	X	X	X	Xing, Jiaqi et al. "Gellan gum/alginate-based Ca-enriched acellular bilayer hydrogel with robust interface bonding for effective osteochondral repair." *Carbohydrate polymers*vol. 270 (2021): 118382.
Biomat only	Monolayer	/	Col + CS	/	/	/	/	/	Molding	Rabbit	X	X				Bauza-Mayol, Guillermo et al. "Biomimetic Scaffolds Modulate the Posttraumatic Inflammatory Response in Articular Cartilage Contributing to Enhanced Neoformation of Cartilaginous Tissue In Vivo." *Advanced healthcare materials*, e2101127. 18 Oct. 2021,
Biomat + biologics	Monolayer	/	CaP	/	/	/	Amino acids	/	Malleable	Sheep	X	X	X	X		Kresakova, Lenka et al. "In Vivo Study of Osteochondral Defect Regeneration Using Innovative Composite Calcium Phosphate Biocement in a Sheep Model." Materials (Basel, Switzerland) vol. 14,16 4471. 10 Aug. 2021, doi:10.3390/ma14164471
Biomat + biologics	Monolayer	/	Gelatin + silk fibroin	/	/	/	SDF-1, TGF-b1	Press-fit	Cut	Rat	X	X	X			Chen, Yuanfeng et al. "Sustained Release SDF-1α/TGF-β1-Loaded Silk Fibroin-Porous Gelatin Scaffold Promotes Cartilage Repair." ACS applied materials & interfaces vol. 11,16 (2019): 14608-14618.
Biomat + biologics	Monolayer	/	PLGA microspheres + agarose	/	/	/	Dexamethasone	Press-fit	Punch	Dog	X	X				Stefani, Robert M et al. "Sustained low-dose dexamethasone delivery via a PLGA microsphere-embedded agarose implant for enhanced osteochondral repair." Acta biomaterialia vol. 102 (2020): 326-340.
Biomat + biologics	Bilayer	Biomat	GelMA	/	GelMA + HAP	/	BMP-2, TGF-b3	/	Molding	Rabbit	X	X				Gan, Donglin et al. "Mussel-Inspired Tough Hydrogel with In Situ Nanohydroxyapatite Mineralization for Osteochondral Defect Repair." Advanced healthcare materials vol. 8,22 (2019): e1901103.
Biomat + biologics	Monolayer	/	Pluronic F-127	/	/	/	Adeno-Associated Viral Vectors	/	Injection	Minipig	X	X	X	X		Madry, Henning et al. "Thermosensitive Hydrogel Based on PEO-PPO-PEO Poloxamers for a Controlled In Situ Release of Recombinant Adeno-Associated Viral Vectors for Effective Gene Therapy of Cartilage Defects." Advanced materials (Deerfield Beach, Fla.) vol. 32,2 (2020): e1906508.
Biomat + biologics	Bilayer	Biomat + biologics	Alginate	/	Graphene oxide	/	Aptamer, kartogenin	/	Other	Rat	X		X	X		Hu, Xiaoxia et al. "A Difunctional Regeneration Scaffold for Knee Repair based on Aptamer-Directed Cell Recruitment." *Advanced materials (Deerfield Beach, Fla.)* vol. 29,15 (2017): 10.1002/adma.201605235.
Biomat + biologics	Monolayer	/	Col I	/	/	/	SDF-1	/	Molding	Rabbit	X	X	X		X	Chen, Pengfei et al. "Radially oriented collagen scaffold with SDF-1 promotes osteochondral repair by facilitating cell homing." Biomaterialsvol. 39 (2015): 114-23.
Biomat + biologics	Monolayer	/	PEOT + PBT	/	/	/	TGF-b1	/	Molding	Minipig	X	X	X	X		Asen, Ann-Kathrin et al. "Sustained spatiotemporal release of TGF-β1 confers enhanced very early chondrogenic differentiation during osteochondral repair in specific topographic patterns." FASEB journal : official publication of the Federation of American Societies for Experimental Biologyvol. 32,10 (2018): 5298-5311.
Biomat + biologics	Monolayer	/	GelMA + cartilage ECM	/	/	/	Exosomes	/	3D-printing	Rabbit	X	X		X		Chen, Pengfei et al. "Desktop-stereolithography 3D printing of a radially oriented extracellular matrix/mesenchymal stem cell exosome bioink for osteochondral defect regeneration." Theranostics vol. 9,9 2439-2459. 13 Apr. 2019,
Biomat + biologics	Monolayer	/	HA + silk fibroin	/	/	/	Aptamer	/	Molding	Rabbit	X	X	X			Wang, Xin et al. "Aptamer-Functionalized Bioscaffold Enhances Cartilage Repair by Improving Stem Cell Recruitment in Osteochondral Defects of Rabbit Knees." *The American journal of sports medicine* vol. 47,10 (2019): 2316-2326.
Biomat + biologics	Monolayer	/	PLGA	/	/	/	PRP	Press-fit	Molding	Rabbit	X	X	X	X		Chang, Nai-Jen et al. "Therapeutic Effects of the Addition of Platelet-Rich Plasma to Bioimplants and Early Rehabilitation Exercise on Articular Cartilage Repair." *The American journal of sports medicine* vol. 46,9 (2018): 2232-2241.

Biomat + biologics	Monolayer	/	Col + HAP	/	/	/	BMP-2	/	Cut	Rabbit	X		X	X		Taniyama, Takashi et al. "Repair of osteochondral defects in a rabbit model using a porous hydroxyapatite collagen composite impregnated with bone morphogenetic protein-2." *Artificial organs* vol. 39,6 (2015): 529-35.
Biomat + biologics	Quadrilayer	Biomat + biologics	Alginate + chitosan	Alginate + chitosan + HAP	Alginate + PCL + PEG + HAP	/	BMP-2, FGF-2, TGF-b1	/	Electrospinning + Molding	Rabbit	X		X	X		Chen, Taijun et al. "A single integrated osteochondral in situ composite scaffold with a multi-layered functional structure." *Colloids and surfaces. B, Biointerfaces* vol. 167 (2018): 354-363.
Biomat + biologics	Monolayer	/	Gelatin	/	/	/	IGF-1	Press-fit	Commercial	Rat	X	X				Alemdar, Celil et al. "Effect of insulin-like growth factor-1 and hyaluronic acid in experimentally produced osteochondral defects in rats." *Indian journal of orthopaedics*vol. 50,4 (2016): 414-20.
Biomat + biologics	Monolayer	/	HA-MA + HAP + PCL + PEG	/	/	/	TGF-b1	/	3D-printing	Minipig	X	X		X		Hsieh, Yi-Ho et al. "Healing of Osteochondral Defects Implanted with Biomimetic Scaffolds of Poly(ε-Caprolactone)/Hydroxyapatite and Glycidyl-Methacrylate-Modified Hyaluronic Acid in a Minipig." *International journal of molecular sciences* vol. 19,4 1125. 9 Apr. 2018
Biomat + biologics	Bilayer	Biomat + biologics	Cellulose + GAG	/	Cellulose + HAP	/	BMP-2, TGF-b3	Press-fit	Undefined	Rat	X	X		X		Kumbhar, Jyoti V et al. "In vitro and in vivo studies of a novel bacterial cellulose-based acellular bilayer nanocomposite scaffold for the repair of osteochondral defects." *International journal of nanomedicine* vol. 12 6437-6459. 1 Sep. 2017,
Biomat + biologics	Monolayer	/		/	Porous tantalum	/	BMP-2	/	Molding	Rabbit	X			X	X	Wang, Qian et al. "Application of combined porous tantalum scaffolds loaded with bone morphogenetic protein 7 to repair of osteochondral defect in rabbits." *International orthopaedics* vol. 42,7 (2018): 1437-1448.
Biomat + biologics	Monolayer	/	Col	/	/	/	TGF-b1	Press-fit	Molding	Rabbit	X		X			Maruki, Hideyuki et al. "Effects of a cell-free method using collagen vitrigel incorporating TGF-β1 on articular cartilage repair in a rabbit osteochondral defect model." *Journal of biomedical materials research. Part B, Applied biomaterials* vol. 105,8 (2017): 2592-2602.
Biomat + biologics	Monolayer	/	Peptide-based hydrogels	/	/	/	IGF-1, PDGF	/	Malleable	Rabbit	X	X	X			Zanotto, Gustavo et al. "Trypsin Pre-Treatment Combined With Growth Factor Functionalized Self-Assembling Peptide Hydrogel Improves Cartilage Repair in Rabbit Model." *Journal of orthopaedic research : official publication of the Orthopaedic Research Society* vol. 37,11 (2019): 2307-2315.
Biomat + biologics	Monolayer	/	Gellan gum	/	/	/	Tenascin-C	/	Punch	Rabbit	X	X	X			Ikemura, Shigeto et al. "Effect of tenascin-C on the repair of full-thickness osteochondral defects of articular cartilage in rabbits." Journal of orthopaedic research : official publication of the Orthopaedic Research Society vol. 33,4 (2015): 563-71.
Biomat + biologics	Bilayer	Biologics	Alginate	/	Alginate	/	BMP-4, TGF-b1	/	Injection	Minipig	X	X	X	X		Ruvinov, Emil et al. "Articular cartilage regeneration using acellular bioactive affinity-binding alginate hydrogel: A 6-month study in a mini-pig model of osteochondral defects." *Journal of orthopaedic translation* vol. 16 40-52. 9 Sep. 2018,
Biomat + biologics	Monolayer	/	Gelatin + silk fibroin	/	/	/	Ginsenoside Rb1, TGF-b1	/	Cut	Rat	X	X	X			Wu, Tingting et al. "Ginsenoside Rb1/TGF-β1 loaded biodegradable silk fibroin-gelatin porous scaffolds for inflammation inhibition and cartilage regeneration." *Materials science & engineering. C, Materials for biological applications* vol. 111 (2020): 110757.
Biomat + biologics	Monolayer	/	Alginate + HA	/	/	/	Berberine	/	Undefined	Rat	X	X	X			Chen, Pengfei et al. "Interpenetrating polymer network scaffold of sodium hyaluronate and sodium alginate combined with berberine for osteochondral defect regeneration." *Materials science & engineering. C, Materials for biological applications* vol. 91 (2018): 190-200.
Biomat + biologics	Bilayer	Biomat	PLGA	/	PLGA	/	PRP	/	Molding	Rabbit	X	X	X	X		Zhang, Yong-Tao et al. "Repair of Osteochondral Defects in a Rabbit Model Using Bilayer Poly(Lactide-co-Glycolide) Scaffolds Loaded with Autologous Platelet-Rich Plasma." Medical science monitor : international medical journal of experimental and clinical research vol. 23 5189-5201. 31 Oct. 2017,
Biomat + biologics	Monolayer	/	HA-MA	/	/	/	Autologous PRP	/	Injection	Minipig	X	X	X	X	X	Yan, Wenqiang et al. "Platelet-rich plasma combined with injectable hyaluronic acid hydrogel for porcine cartilage regeneration: a 6-month follow-up." Regenerative biomaterialsvol. 7,1 (2020): 77-90.
Biomat + biologics	Bilayer	/	CS + PLGA	/	b-TCP + PLGA	/	IGF-1, TGF-b3	Press-fit	Molding	Sheep	X	X			X	Mohan, Neethu et al. "Microsphere-based gradient implants for osteochondral regeneration: a long-term study in sheep." *Regenerative medicine* vol. 10,6 (2015): 709-28.
Biomat + biologics	Bilayer	Biomat + biologics	Gelatin + OPF	/	Gelatin + OPF	/	BMP-2, IGF-1	/	Molding	Rabbit	X			X		Lu, Steven et al. "Technical Report: Correlation Between the Repair of Cartilage and Subchondral Bone in an Osteochondral Defect Using Bilayered, Biodegradable Hydrogel Composites." *Tissue engineering. Part C, Methods* vol. 21,12 (2015): 1216-25.
Biomat + biologics	Trilayer	Biomat + biologics	CS + glycidyl-MA + PEGD-MA	CS + glycidyl-MA + PEGD-MA	CS + glycidyl-MA + PEGD-MA+ octoviny polyhedral oligomeric silsesquioxanes	/	TGF-b1	/	Molding	Rabbit	X	X				Han, Fengxuan et al. "Photocrosslinked layered gelatin-chitosan hydrogel with graded compositions for osteochondral defect repair." Journal of materials science. Materials in medicine vol. 26,4 (2015): 160.
Biomat + biologics	Bilayer	Biomat	PLCL	/	b-TCP + PLA	/	IL-8, MIP-3a	Press-fit	Molding	Dog	X	X		X		Park, Min Sung et al. "In Situ Recruitment of Human Bone Marrow-Derived Mesenchymal Stem Cells Using Chemokines for Articular Cartilage Regeneration." Cell transplantation vol. 24,6 (2015): 1067-83.
Biomat + biologics	Monolayer	/	Dentin ECM			/	Alendronate	/	Malleable	Rabbit	X	X		X		Yasuda, Atsushi et al. "Experimental study of influence of direct bisphosphonate administration on bone substitute." Dental materials journal vol. 34,6 (2015): 789-95.
Biomat + biologics	Trilayer	Biomat	Alginate + chitosan + polyelectrolyte	Alginate + b-TCP + chitosan + polyelectrolyte	Alginate + b-TCP + chitosan + polyelectrolyte	/	Dexamethasone	/	Molding	Rat	X					Algul, Derya et al. "In vitro release and In vivo biocompatibility studies of biomimetic multilayered alginate-chitosan/β-TCP scaffold for osteochondral tissue." Journal of biomaterials science. Polymer edition vol. 27,5 (2016): 431-40.
Biomat + biologics	Monolayer	/	Chitosan	/	/	/	Peptides	Press-fit	Undefined	Rabbit	X	X	X			Chen, Jiaqing et al. "TGF-β1 affinity peptides incorporated within a chitosan sponge scaffold can significantly enhance cartilage regeneration." Journal of materials chemistry. B vol. 6,4 (2018): 675-687.
Biomat + biologics	Monolayer	/	PRF + cartilage fragments	/	/	/	PRF	Press-fit + periostal flap	Malleable	Minipig	X	X			X	Sheu, S Y et al. "The effect of platelet-rich fibrin on autologous osteochondral transplantation: An in vivo porcine model." *The Knee* vol. 24,6 (2017): 1392-1401.
Biomat + biologics	Monolayer	/	PRF	/	/	/	PRF	/	Injection	Rabbit	X	X				Abd El Raouf, Mustafa et al. "Injectable-platelet rich fibrin using the low speed centrifugation concept improves cartilage regeneration when compared to platelet-rich plasma." *Platelets* vol. 30,2 (2019): 213-221.
Biomat + biologics	Bilayer	Biomat + biologics	Glycidyl acid-MA + HA-MA + PCL + PEG	/	HAP + PCL + PEG	/	TGF-b1	/	3D-printing	Rabbit	X					Hsieh, Yi-Ho et al. "Osteochondral Regeneration Induced by TGF-β Loaded Photo Cross-Linked Hyaluronic Acid Hydrogel Infiltrated in Fused Deposition-Manufactured Composite Scaffold of Hydroxyapatite and Poly (Ethylene Glycol)-Block-Poly(ε-Caprolactone)." *Polymers* vol. 9,5 182. 20 May. 2017,
Biomat + biologics	Bilayer	Biomat + biologics	PNAGA	/	PNAGA + b-TCP	/	TGF-b1	/	3D-printing	Rat	X		X	X		Gao, Fei., et al. "Direct 3D Printing of High Strength Biohybrid Gradient Hydrogel Scaffolds for Efficient Repair of Osteochondral Defect." Advanced Functional Materials, 28(13), (2018):1706644.
Biomat + biologics	Bilayer	Biomat + biologics	Alginate + glycol chitosan + PEO	/	Alginate + BG + glycol chitosan + PEO	/	TGF-b1	/	Molding	Rabbit	X	X	X	X		Liu, Bingchuan et al. "Biphasic Double-Network Hydrogel With Compartmentalized Loading of Bioactive Glass for Osteochondral Defect Repair." *Frontiers in bioengineering and biotechnology* vol. 8 752. 2 Jul. 2020,
Biomat + biologics	Monolayer	/	Col	/	/	/	Wnt5a	/	Molding	Rabbit	X	X	X			Qi, Yiying et al. "An oriented-collagen scaffold including Wnt5a promotes osteochondral regeneration and cartilage interface integration in a rabbit model." FASEB journal : official publication of the Federation of American Societies for Experimental Biology vol. 34,8 (2020): 11115-11132.
Biomat + biologics	Monolayer	/	Alginate	/	/	/	Peptides	/	Undefined	Rabbit	X	X		X		Wei Zhang et al. "Tannic acid-mediated dual peptide-functionalized scaffolds to direct stem cell behavior and osteochondral regeneration." Chemical Engineering Journal, 396, (2020) 125232.
Biomat + biologics	Monolayer	/	Cartilage ECM + PEG	/	/	/	Honokiol	/	3D-printing	Rat	X	X	X	X		Zhu, Shouan et al. "3D-Printed Extracellular Matrix/Polyethylene Glycol Diacrylate Hydrogel Incorporating the Anti-inflammatory Phytomolecule Honokiol for Regeneration of Osteochondral Defects." The American journal of sports medicine vol. 48,11 (2020): 2808-2818.
Biomat + biologics	Monolayer	/	Col I	/	/	/	Agrin	/	Injection (mouse) Malleable (sheep)	Mouse, sheep	X			X		Eldridge, Suzanne E et al. "Agrin induces long-term osteochondral regeneration by supporting repair morphogenesis." *Science translational medicine* vol. 12,559 (2020): eaax9086.
Biomat + biologics	Monolayer	/	Silk fibroin	/	/	/	Kartogenin, peptides	/	Undefined	Rabbit	X	X	X	X		Zhang, Wei et al. "An all-silk-derived functional nanosphere matrix for sequential biomolecule delivery and *in situ* osteochondral regeneration." *Bioactive materials* vol. 5,4 832-843. 23 Jun. 2020,
Biomat + biologics	Monolayer	/	AH + PLGA	/	/	/	Kartogenin	/	Injection	Minipig	X	X	X	X	X	Yan, Wenqiang et al. "An Injectable Hydrogel Scaffold With Kartogenin-Encapsulated Nanoparticles for Porcine Cartilage Regeneration: A 12-Month Follow-up Study." *The American journal of sports medicine* vol. 48,13 (2020): 3233-3244.
Biomat + biologics	Bilayer	Biomat + biologics	GelMA	/	HAP + PCL	/	IL-4	/	3D-printing	Rabbit	X	X	X	X	X	Gong, Lin et al. "An interleukin-4-loaded bi-layer 3D printed scaffold promotes osteochondral regeneration." *Acta biomaterialia* vol. 117 (2020): 246-260.
Biomat + biologics	Monolayer	/	Chitosan	/	/	/	Icariin-conditioned serum	/	Injection	Rabbit	X	X	X			Zhang, Juntao et al. "Repair of osteochondral defect using icariin-conditioned serum combined with chitosan in rabbit knees." *BMC complementary medicine and therapies* vol. 20,1 193. 22 Jun. 2020,
Biomat + biologics	Monolayer	/	HA	/	/	/	Adipose tissue-derived stromal vascular fraction	/	Cut	Rabbit	X	X				Şahin, Abdullah Alper et al. "Effects of adipose tissue-derived stromal vascular fraction on osteochondral defects treated by hyaluronic acid-based scaffold: An experimental study." *Joint diseases and related surgery* vol. 32,2 (2021): 347-354.
Biomat + biologics	Monolayer	/	Col + gelatin	/	/	/	SDF-1	/	Commercial	Rat	X					Mustapich, Taylor et al. "A Novel Strategy to Enhance Microfracture Treatment With Stromal Cell-Derived Factor-1 in a Rat Model." *Frontiers in cell and developmental biology* vol. 8 595932. 4 Feb. 2021,
Biomat + biologics	Monolayer	/	PRP	/	/	/	PRP	/	Malleable	Rabbit	X	X	X			Slimi, Fathia et al. "The effectiveness of platelet-rich plasma gel on full-thickness cartilage defect repair in a rabbit model." *Bone & joint research* vol. 10,3 (2021): 192-202.

Biomat + biologics	Monolayer	/	Alginate	/	/	/	rAAV-mediated IGF-I	/	Undefined	Minipig	X	X	X			Maihöfer, Johanna et al. "Hydrogel-Guided, rAAV-Mediated IGF-I Overexpression Enables Long-Term Cartilage Repair and Protection against Perifocal Osteoarthritis in a Large-Animal Full-Thickness Chondral Defect Model at One Year In Vivo." *Advanced materials (Deerfield Beach, Fla.)* vol. 33,16 (2021): e2008451.
Biomat + biologics	Monolayer	/	GelMA	/	/	/	Histatin-1	/	Injection	Rabbit	X	X	X			Shi, Changjing et al. "Human Salivary Histatin-1-Functionalized Gelatin Methacrylate Hydrogels Promote the Regeneration of Cartilage and Subchondral Bone in Temporomandibular Joints." Pharmaceuticals (Basel, Switzerland) vol. 14,5 484. 19 May. 2021,
Biomat + biologics	Monolayer	/	Cartilage ECM	/	/	/	IGF-1	/	Undefined	Rabbit	X	X		X		Das, Piyali et al. "Decellularized xenogenic cartilage extracellular matrix (ECM) scaffolds for the reconstruction of osteochondral defects in rabbits." *Journal of materials chemistry. B* vol. 9,24 (2021): 4873-4894
Biomat + biologics	Monolayer	/	Cartilage ECM	/	/	/	IL-4	/	Molding	Rat	X	X	X			Tian, Guangzhao et al. "Cell-free decellularized cartilage extracellular matrix scaffolds combined with interleukin 4 promote osteochondral repair through immunomodulatory macrophages: In vitro and in vivo preclinical study." *Acta biomaterialia* vol. 127 (2021): 131-145.
Biomat + biologics	Monolayer	/	Peptide-based hydrogels	/	/	/	IGF-1, PDGF	/	Malleable	Horse	X	X	X		X	Zanotto, Gustavo M et al. "Microfracture Augmentation With Trypsin Pretreatment and Growth Factor-Functionalized Self-assembling Peptide Hydrogel Scaffold in an Equine Model." The American journal of sports medicine vol. 49,9 (2021): 2498-2508.
Biomat + biologics	Monolayer	/	GelMA	/	/	/	PRP	/	Undefined	Rabbit	X	X	X	X		Jiang, Guangyao et al. "A 3D-printed PRP-GelMA hydrogel promotes osteochondral regeneration through M2 macrophage polarization in a rabbit model." Acta biomaterialia vol. 128 (2021): 150-162.
Biomat + biologics	Bilayer	Biomat	β-glycérophosphate + chitosan	Hydroxide chitosan	/	/	Peptides	/	Molding	Rabbit	X	X	X	X		Liu, Pei.,"Biphasic CK2.1-coated β-glycerophosphate chitosan/LL37-modified layered double hydroxide chitosan composite scaffolds enhance coordinated hyaline cartilage and subchondral bone regeneration." Chemical Engineering Journal, 418,(2021) 129531.
Biomat + biologics	Monolayer	/	Cartilage ECM	/	/	/	Exosomes	/	Molding	Rabbit	X	X	X	X	X	Jiang, Shuangpeng et al. "Enhancement of acellular cartilage matrix scaffold by Wharton's jelly mesenchymal stem cell-derived exosomes to promote osteochondral regeneration." Bioactive materials vol. 6,9 2711-2728. 13 Feb. 2021,
Biomat + biologics	Monolayer	/	PLGA+ PCL	/	/	/	Insulin	/	3D-printing	Rabbit	X	X	X	X		Continued sustained insulin-releasing PLGA naNoparticles modified 3D-Printed PCL composite scaffolds for osteochondral repair
Biomat + biologics	Trilayer	Biomat	PLLA + GelMA + HA + CS	PLLA + GelMA	Apatite + GelMA+ PLLA	/	Peptides	/	Electrospinning	Rabbit	X	X	X	X	X	Gradient fibrous aerogel conjugated with chemokine peptide for regulating cell differentiation and facilitating osteochondral regeneration
Biomat + cells	Bilayer	Biomat	PEG + PGA + PNIPAAm + peptide	/	PEG + PGA + PNIPAAm + peptide	BM-MSCs	/	/	Molding	Rabbit	X	X				Guo, Jason L et al. "Bilayered, peptide-biofunctionalized hydrogels for in vivo osteochondral tissue repair." *Acta biomaterialia* vol. 128 (2021): 120-129.
Biomat + cells	Monolayer	/	Cartilage ECM	/	/	Articular chondrocytes / Adip-MSCs	/	Fibrin glue	Other	Rabbit	X	X	X	X	X	Yin, Heyong et al. "Functional tissue-engineered microtissue derived from cartilage extracellular matrix for articular cartilage regeneration." Acta biomaterialia vol. 77 (2018): 127-141.
Biomat + cells	Trilayer	Biomat	Cartilage ECM	PLGA + b-TCP	PLGA + b-TCP + Col	BM-MSCs	/	/	Molding + 3D-printing	Goat	X	X			X	Zhang, Ting et al. "Biomimetic design and fabrication of multilayered osteochondral scaffolds by low-temperature deposition manufacturing and thermal-induced phase-separation techniques." *Biofabrication* vol. 9,2 025021. 23 May. 2017,
Biomat + cells	Monolayer	/	CS + PEG	/	/	BM-MSCs	/	/	Molding	Rabbit	X	X	X	X		Feng, Xue et al. "Enhanced regeneration of osteochondral defects by using an aggrecanase-1 responsively degradable and N-cadherin mimetic peptide-conjugated hydrogel loaded with BMSCs." Biomaterials science vol. 8,8 (2020): 2212-2226
Biomat + cells	Monolayer	/	Chitosan-Si + HPMC-Si	/	/	Adip-MSCs	/	/	Injection	Dog	X	X	X			Boyer, Cécile et al. "A Self-Setting Hydrogel of Silylated Chitosan and Cellulose for the Repair of Osteochondral Defects: From *in vitro* Characterization to Preclinical Evaluation in Dogs." *Frontiers in bioengineering and biotechnology* vol. 8 23. 29 Jan. 2020,
Biomat + cells	Monolayer	/	HA	/	/	UCB-MSCs	/	/	Malleable	Minipig	X	X	X			Ha, Chul-Won et al. "Cartilage Repair Using Composites of Human Umbilical Cord Blood-Derived Mesenchymal Stem Cells and Hyaluronic Acid Hydrogel in a Minipig Model." *Stem cells translational medicine* vol. 4,9 (2015):
Biomat + cells	Monolayer	/	Cartilage ECM	/	/	Adip-MSCs	/	/	Molding	Rabbit	X	X				Li, Xu et al. "Enrichment of CD146+ Adipose-Derived Stem Cells in Combination with Articular Cartilage Extracellular Matrix Scaffold Promotes Cartilage Regeneration." Theranostics vol. 9,17 5105-5121. 9 Jul. 2019
Biomat + cells	Monolayer	/	Col I	/	/	BM-MSCs	/	/	Other	Macaque	X	X	X			Araki, Susumu et al. "Improved quality of cartilage repair by bone marrow mesenchymal stem cells for treatment of an osteochondral defect in a cynomolgus macaque model." *Acta orthopaedica* vol. 86,1 (2015): 119-26.
Biomat + cells	Monolayer	/	Alginate + OC grafts	/	/	BM-MSCs expressing TGF-b	/	Alginate gel	Cut + malleable	Goat	X	X				Sun, Jun et al. "Restore a 9 mm diameter osteochondral defect with gene enhanced tissue engineering followed mosaicplasty in a goat model." *Acta orthopaedica et traumatologica turcica* vol. 50,4 (2016): 464-9.
Biomat + cells	Bilayer	Cells	Fibrin	/	Fibrin	BM-MSCs	/	/	Molding	Rabbit	X		X		X	Wu, Yingnan et al. "Repair of Osteochondral Defects With Predifferentiated Mesenchymal Stem Cells of Distinct Phenotypic Character Derived From a Nanotopographic Platform." *The American journal of sports medicine* vol. 48,7 (2020): 1735-1747.
Biomat + cells	Monolayer	/	Cartilage particles + Fibrin	/	/	Amniotic MSCs	/	/	Other	Rabbit	X	X	X			You, Qi et al. "Human Amniotic Mesenchymal Stem Cell Sheets Encapsulating Cartilage Particles Facilitate Repair of Rabbit Osteochondral Defects." *The American journal of sports medicine* vol. 48,3 (2020): 599-611
Biomat + cells	Monolayer	/	Cartilage particles + Col	/	/	Articular chondrocytes	/	Periostal flap	Malleable	Rabbit	X	X	X			Matsushita, Ryosuke et al. "Repair of an Osteochondral Defect With Minced Cartilage Embedded in Atelocollagen Gel: A Rabbit Model." *The American journal of sports medicine* vol. 47,9 (2019): 2216-2224.
Biomat + cells	Monolayer	/	CS + PEG	/	/	BM-MSCs	/	Defect drying with CO2	Injection	Rabbit	X	X	X			Pascual-Garrido, Cecilia et al. "Photopolymerizable Injectable Cartilage Mimetic Hydrogel for the Treatment of Focal Chondral Lesions: A Proof of Concept Study in a Rabbit Animal Model." *The American journal of sports medicine* vol. 47,1 (2019): 212-221.
Biomat + cells	Monolayer	/	HA	/	/	UBC-MSCs (differentiated into chondrocytes or undifferentiated	/	/	Malleable	Rat	X	X	X			Park, Yong-Beom et al. "Comparison of Undifferentiated Versus Chondrogenic Predifferentiated Mesenchymal Stem Cells Derived From Human Umbilical Cord Blood for Cartilage Repair in a Rat Model." *The American journal of sports medicine* vol. 47,2 (2019): 451-461.
Biomat + cells	Monolayer	/	Fibrin	/	/	Articular chondrocytes (dediff or dediff et redifferentiated)	/	Fibrin glue + press-fit	Malleable	Rabbit	X		X			Bianchi, Vanessa J et al. "Redifferentiated Chondrocytes in Fibrin Gel for the Repair of Articular Cartilage Lesions." *The American journal of sports medicine* vol. 47,10 (2019): 2348-2359.
Biomat + cells	Monolayer	/	Col II	/	/	Articular chondrocytes	/	/	Undefined	Rabbit	X	X				Wong, Chin-Chean et al. "Facilitating In Vivo Articular Cartilage Repair by Tissue-Engineered Cartilage Grafts Produced From Auricular Chondrocytes." *The American journal of sports medicine* vol. 46,3 (2018): 713-727.
Biomat + cells	Monolayer	/	Fibrin	/	/	BM-MSCs	/	/	Malleable	Rabbit	X	X				Lee, Byung Hoon et al. "Therapeutic Efficacy of Spherical Aggregated Human Bone Marrow-Derived Mesenchymal Stem Cells Cultured for Osteochondral Defects of Rabbit Knee Joints." *The American journal of sports medicine* vol. 46,9 (2018): 2242-2252.
Biomat + cells	Bilayer	Biomat + cells		/	b-TCP or HAP	Syn-MSCs	/	Press-fit	Commercial	Rabbit	X				X	Shimomura, Kazunori et al. "Comparison of 2 Different Formulations of Artificial Bone for a Hybrid Implant With a Tissue-Engineered Construct Derived From Synovial Mesenchymal Stem Cells: A Study Using a Rabbit Osteochondral Defect Model." The American journal of sports medicine vol. 45,3 (2017): 666-675.
Biomat + cells	Monolayer	/	PCL + PLA	/	/	BM-MSCs	/	Press-fit	Other	Rabbit	X	X	X			Barron, Valerie et al. "Evaluation of the Early In Vivo Response of a Functionally Graded Macroporous Scaffold in an Osteochondral Defect in a Rabbit Model." *Annals of biomedical engineering* vol. 44,5 (2016): 1832-44.
Biomat + cells	Monolayer	/	PEOT + PBT	/	/	Other MSCs	/	Press-fit	3D-printing	Rabbit	X		X			Barron, V et al. "Evaluation of Cartilage Repair by Mesenchymal Stem Cells Seeded on a PEOT/PBT Scaffold in an Osteochondral Defect." Annals of biomedical engineering vol. 43,9 (2015): 2069-82.
Biomat + cells	Monolayer	/	Cartilage ECM	/	/	UCB-MSCs (differentiated into chondrocytes or undifferentiated)	/	/	Molding	Rabbit	X	X				Liu, Shuyun et al. "Repair of Osteochondral Defects Using Human Umbilical Cord Wharton's Jelly-Derived Mesenchymal Stem Cells in a Rabbit Model." *BioMed research international* vol. 2017 (2017): 8760383
Biomat + cells	Monolayer	/	Chitosan + PVA	/	/	BM-MSCs	/	Bio-protein glue	Cut	Rabbit	X	X	X			Peng, Liangquan et al. "Characterization of a novel polyvinyl alcohol/chitosan porous hydrogel combined with bone marrow mesenchymal stem cells and its application in articular cartilage repair." *BMC musculoskeletal disorders* vol. 20,1 257. 29 May. 2019,
Biomat + cells	Bilayer	Biomat + cells	Col II + b-TCP	/	Col I + b-TCP	BM-MSCs	/	Press-fit	Molding	Dog	X	X	X			Lv, Y M, and Q S Yu. "Repair of articular osteochondral defects of the knee joint using a composite lamellar scaffold." *Bone & joint research* vol. 4,4 (2015): 56-64.
Biomat + cells	Trilayer	Biomat	Silicon-zirconium + hybrid organic-inorganic polymer	Silicon-zirconium + hybrid organic-inorganic polymer	Silicon-zirconium + hybrid organic-inorganic polymer	Articular chondrocytes	/	Fibrin clot	Other	Rabbit	X	X			X	Maciulaitis, Justinas et al. "Osteochondral Repair and Electromechanical Evaluation of Custom 3D Scaffold Microstructured by Direct Laser Writing Lithography." *Cartilage* vol. 13,2_suppl (2021): 615S-625S.
Biomat + cells	Bilayer	Biomat	Col I	/	b-TCP + PLA	Mixed cells (from bone marrow aspirate)	/	/	Cut	Horse	X	X	X		X	McCarrel, Taralyn M et al. "Two-Year Evaluation of Osteochondral Repair with a Novel Biphasic Graft Saturated in Bone Marrow in an Equine Model." *Cartilage* vol. 8,4 (2017): 406-416.
Biomat + cells	Monolayer	/	Alginate	/	/	Nasal chondrocytes	/	/	Injection	Rabbit	X			X	X	Chen, Wenliang et al. "Autologous nasal chondrocytes delivered by injectable hydrogel for in vivo articular cartilage regeneration." *Cell and tissue banking* vol. 19,1 (2018): 35-46.
Biomat + cells	Monolayer	/	Fibrin + silk fibroin	/	/	Articular chondrocytes	/	/	Molding	Rabbit	X	X	X		X	Kazemnejad, Somaieh et al. "Comparative repair capacity of knee osteochondral defects using regenerated silk fiber scaffolds and fibrin glue with/without autologous chondrocytes during 36 weeks in rabbit model." *Cell and tissue research* vol. 364,3 (2016): 559-572.

Biomat + cells	Monolayer	/	Col + TCP	/	/	BM-MSCs	/	/	Molding	Rabbit	X	X	X			Meng, F G et al. "Chondrogenesis of mesenchymal stem cells in a novel hyaluronate-collagen-tricalcium phosphate scaffolds for knee repair." *European cells & materials* vol. 31 79-94. 5 Jan. 2016
Biomat + cells	Monolayer	/	Demineralized bone ECM + gellan gum	/	/	Articular chondrocytes	/	/	Punch	Rabbit	X			X		Kim, David et al. "Osteochondral and bone tissue engineering scaffold prepared from Gallus var domesticus derived demineralized bone powder combined with gellan gum for medical application." International journal of biological macromolecules vol. 149 (2020): 381-394.
Biomat + cells	Monolayer	/	Chitosan + HA	/	/	Articular chondrocytes	/	Press-fit	Undefined	Rabbit	X	X	X			Mohan, Neethu et al. "Chitosan-hyaluronic acid hydrogel for cartilage repair." International journal of biological macromolecules vol. 104,Pt B (2017): 1936-1945.
Biomat + cells	Bilayer	Biomat + cells	Cartilage ECM + silk fibroin	/	Bone ECM	Adip-MSCs	/	Tisuacryl glue	Molding	Minipig	X	X	X			Pérez-Silos, Vanessa et al. "A Cellularized Biphasic Implant Based on a Bioactive Silk Fibroin Promotes Integration and Tissue Organization during Osteochondral Defect Repair in a Porcine Model." International journal of molecular sciences vol. 20,20 5145. 17 Oct. 2019,
Biomat + cells	Monolayer	/	PLGA	/	/	Other cells (Endothelial Progenitor Cells)	/	Press-fit	Molding	Rabbit	X	X	X	X		Wang, Hsueh-Chun et al. "Continuous Passive Motion Promotes and Maintains Chondrogenesis in Autologous Endothelial Progenitor Cell-Loaded Porous PLGA Scaffolds during Osteochondral Defect Repair in a Rabbit Model." International journal of molecular sciences vol. 20,2 259. 10 Jan. 2019,
Biomat + cells	Bilayer	Cells	PCL	/	PCL	Articluar chondrocytes (cartilage) BM-MSCs (bone)	/	Press-fit	Molding	Minipig	X	X	X	X		Bothe, Friederike et al. "Treatment of Focal Cartilage Defects in Minipigs with Zonal Chondrocyte/Mesenchymal Progenitor Cell Constructs." International journal of molecular sciences vol. 20,3 653. 2 Feb. 2019,
Biomat + cells	Bilayer	Biomat	Chitosan + silk fibroin	/	Chitosan + HAP + silk fibroin	BM-MSCs	/	/	Other	Rabbit		X	X	X		Ruan, Shi-Qiang et al. "Preparation of a biphase composite scaffold and its application in tissue engineering for femoral osteochondral defects in rabbits." International orthopaedics vol. 41,9 (2017): 1899-1908.
Biomat + cells	Bilayer	Biomat	Gelatin + PVA	/	HAP + PA6	BM-MSCs	/	/	Molding	Rabbit	X	X				Li, Xiang et al. "Osteogenesis and chondrogenesis of biomimetic integrated porous PVA/gel/V-n-HA/pa6 scaffolds and BMSCs construct in repair of articular osteochondral defect." Journal of biomedical materials research. Part A vol. 103,10 (2015): 3226-36.
Biomat + cells	Bilayer	Biomat	Col I + HA + PLA-PCL	/	b-TCP	BM-MSCs	/	Press-fit	Cut	Rabbit	X	X	X			Liu, Shen et al. "Osteochondral regeneration using an oriented nanofiber yarn-collagen type I/hyaluronate hybrid/TCP biphasic scaffold." Journal of biomedical materials research. Part A vol. 103,2 (2015): 581-92.
Biomat + cells	Bilayer	Biomat	Cartilage ECM	/	Gelatin + PLGA	Articular chondrocytes	/	Press-fit	Molding	Rabbit	X		X			Nie, Xiaolei et al. "Engineering a multiphasic, integrated graft with a biologically developed cartilage-bone interface for osteochondral defect repair." Journal of materials chemistry. B vol. 7,42 (2019): 6515-6525.
Biomat + cells	Monolayer	/	Col I	/	/	Articular chondrocytes	/	Col I gel	Malleable	Rabbit	X	X	X	X		Yu, Cheng et al. "Repair of osteochondral defects in a rabbit model with artificial cartilage particulates derived from cultured collagen-chondrocyte microspheres." Journal of materials chemistry. B vol. 6,31 (2018): 5164-5173.
Biomat + cells	Monolayer	/	Placenta-derived ECM	/	/	Amniotic MSCs	/	/	Molding	Rabbit	X	X	X			Rameshbabu, Arun Prabhu et al. "Investigating the potential of human placenta-derived extracellular matrix sponges coupled with amniotic membrane-derived stem cells for osteochondral tissue engineering." Journal of materials chemistry. B vol. 4,4 (2016): 613-625.
Biomat + cells	Monolayer	/	Peptide-based hydrogels	/	/	Other MSCs	/	/	Injection	Rabbit	X	X				Pescador, David et al. "Regeneration of hyaline cartilage promoted by xenogeneic mesenchymal stromal cells embedded within elastin-like recombinamer-based bioactive hydrogels." *Journal of materials science. Materials in medicine* vol. 28,8 (2017): 115.
Biomat + cells	Monolayer	/	Acellular dermal ECM	/	/	Adip-MSCs (infrapatellar fat pad)	/	Fibrin glue	Punch	Rabbit	X	X	X			Ye, Ken et al. "Osteochondral repair using an acellular dermal matrix-pilot in vivo study in a rabbit osteochondral defect model." Journal of orthopaedic research : official publication of the Orthopaedic Research Society vol. 36,7 (2018): 1919-1928.
Biomat + cells	Bilayer	Biomat	PLGA	/	PLGA	BM-MSCs	/	Press-fit	Molding	Rabbit	X	X	X		X	Duan, Pingguo et al. "Restoration of osteochondral defects by implanting bilayered poly(lactide-co-glycolide) porous scaffolds in rabbit joints for 12 and 24 weeks." Journal of orthopaedic translation vol. 19 68-80. 17 May. 2019,
Biomat + cells	Monolayer	/	Chitosan + PEO	/	/	Syn-MSCs	/	/	Injection	Rabbit	X	X	X			Jia, Zhaofeng et al. "Repair of osteochondral defects using injectable chitosan-based hydrogel encapsulated synovial fluid-derived mesenchymal stem cells in a rabbit model." Materials science & engineering. C, Materials for biological applications vol. 99 (2019): 541-551.
Biomat + cells	Monolayer	/	/	/	HAP + PCL	UCB-MSCs + articular chondrocytes	/	/	3D-printing	Rabbit	X	X				Zheng, Pengfei et al. "A Rabbit Model of Osteochondral Regeneration Using Three-Dimensional Printed Polycaprolactone-Hydroxyapatite Scaffolds Coated with Umbilical Cord Blood Mesenchymal Stem Cells and Chondrocytes." Medical science monitor : international medical journal of experimental and clinical research vol. 25 7361-7369. 1 Oct. 2019,
Biomat + cells	Monolayer	/	Col I	/	/	Articular chondrocytes	/	/	Cut	Rabbit	X	X				Endo, J et al. "Utility of T2 mapping and dGEMRIC for evaluation of cartilage repair after allograft chondrocyte implantation in a rabbit model." Osteoarthritis and cartilage vol. 23,2 (2015): 280-8.
Biomat + cells	Monolayer	/	HA	/	/	UCB-MSCs	/	/	Malleable	Rabbit	X	X	X			Park, Yong-Beom et al. "Restoration of a large osteochondral defect of the knee using a composite of umbilical cord blood-derived mesenchymal stem cells and hyaluronic acid hydrogel: a case report with a 5-year follow-up." BMC musculoskeletal disorders vol. 18,1 59. 2 Feb. 2017,
Biomat + cells	Monolayer	/	Col I	/	/	BM-MSCs	/	Press-fit	Undefined	Rabbit	X					Fekrazad, Reza et al. "Effects of Photobiomodulation and Mesenchymal Stem Cells on Articular Cartilage Defects in a Rabbit Model." Photomedicine and laser surgery vol. 34,11 (2016): 543-549.
Biomat + cells	Monolayer	/	HA	/	/	UCB-MSCs	/	/	Undefined	Rabbit	X	X	X			Park, Yong-Beom et al. "Effect of Transplanting Various Concentrations of a Composite of Human Umbilical Cord Blood-Derived Mesenchymal Stem Cells and Hyaluronic Acid Hydrogel on Articular Cartilage Repair in a Rabbit Model." PloS one vol. 11,11 e0165446. 8 Nov. 2016,
Biomat + cells	Bilayer	Biomat	Col + GAG	/	CaP + Col + GAG	BM-MSCs + other MSCs (platelet derived MSCs)	/	/	Undefined	Sheep	X	X	X		X	Hopper, Niina et al. "Peripheral Blood Mononuclear Cells Enhance Cartilage Repair in in vivo Osteochondral Defect Model." PloS one vol. 10,8 e0133937. 7 Aug. 2015,
Biomat + cells	Monolayer	/	Peptide-based hydrogels	/	/	BM-MSCs	/	/	Injection	Rabbit	X	X				Cipriani, Filippo et al. "An elastin-like recombinamer-based bioactive hydrogel embedded with mesenchymal stromal cells as an injectable scaffold for osteochondral repair." Regenerative biomaterials vol. 6,6 (2019): 335-347.
Biomat + cells	Bilayer	Biomat	PLGA	/	PLGA	BM-MSCs	/	Press-fit	Molding	Rabbit	X	X	X			Pan, Zhen et al. "Effect of porosities of bilayered porous scaffolds on spontaneous osteochondral repair in cartilage tissue engineering." Regenerative biomaterials vol. 2,1 (2015): 9-19.
Biomat + cells	Monolayer	/	OPF	/	/	Adip-MSCs	/	Press-fit	Punch	Minipig	X		X		X	De Girolamo, Laura et al. "Repair of osteochondral defects in the minipig model by OPF hydrogel loaded with adipose-derived mesenchymal stem cells." Regenerative medicine vol. 10,2 (2015): 135-51.
Biomat + cells	Bilayer	Cells	Col + Porous tantalum	/	Col + Porous tantalum	Articular chondrocytes + BM-MSCs	/	Fibrin glue	Undefined	Goat	X	X	X			Wei, Xiaowei et al. "Mesenchymal stem cell-loaded porous tantalum integrated with biomimetic 3D collagen-based scaffold to repair large osteochondral defects in goats." Stem cell research & therapy vol. 10,1 72. 5 Mar. 2019,
Biomat + cells	Monolayer	/	HA-MA	/	/	BM-MSCs	/	Press-fit	Molding	Rat	X	X	X			Lin, Sien et al. "Synergistic effects on mesenchymal stem cell-based cartilage regeneration by chondrogenic preconditioning and mechanical stimulation." Stem cell research & therapy vol. 8,1 221. 3 Oct. 2017,
Biomat + cells	Monolayer	/	Agarose	/	/	BM-MSCs or Syn-MSCs	/	/	Undefined	Rat	X	X	X			Zayed, Mohammed et al. "Xenogenic Implantation of Equine Synovial Fluid-Derived Mesenchymal Stem Cells Leads to Articular Cartilage Regeneration." Stem cells international vol. 2018 1073705. 6 Jun. 2018,
Biomat + cells	Bilayer	Structure + Biomat	Col I + Col III	/	Col I + Col III	Syn-MSCs or articular chondrocytes	/	Press-fit + Fibrin glue	Commercial	Rabbit	X		X		X	Schmal, Hagen et al. "Comparison of Regenerative Tissue Quality following Matrix-Associated Cell Implantation Using Amplified Chondrocytes Compared to Synovium-Derived Stem Cells in a Rabbit Model for Cartilage Lesions." Stem cells international vol. 2018 4142031. 19 Apr. 2018,
Biomat + cells	Monolayer	/	Fibrin	/	/	Other MSCs (embryonic stem cells)	/	/	Cut	Rat	X	X				Wang, Tao et al. "Enhanced chondrogenesis from human embryonic stem cells." Stem cell research vol. 39 (2019): 101497.
Biomat + cells	Monolayer	/	Alginate	/	/	BM-MSCs	/	Press-fit	Molding	Rabbit	X		X	X	X	Critchley, Susan et al. "Regeneration of Osteochondral Defects Using Developmentally Inspired Cartilaginous Templates." Tissue engineering. Part A vol. 25,3-4 (2019): 159-171
Biomat + cells	Trilayer	Biomat + cells	Gelatin + chitosan	Gelatin + chitosan	b-TCP	BM-MSCs + chondrocytes	/	Press-fit	Molding + Bioreactor	Goat	X	X	X			Zhai, Chenjun et al. "Repair of Articular Osteochondral Defects Using an Integrated and Biomimetic Trilayered Scaffold." Tissue engineering. Part A vol. 24,21-22 (2018): 1680-1692.
Biomat + cells	Monolayer	/	Gelatin	/	/	Articular chondrocytes	/	/	Cut	Rabbit	X	X	X		X	Wang, Chen-Chie et al. "Expandable Scaffold Improves Integration of Tissue-Engineered Cartilage: An In Vivo Study in a Rabbit Model." Tissue engineering. Part A vol. 22,11-12 (2016): 873-84.
Biomat + cells	Bilayer	Biomat + cells	Col I	/	HAP	Articular chondrocytes	/	/	Molding	Pork	X	X	X		X	Sosio, Corrado et al. "Osteochondral repair by a novel interconnecting collagen-hydroxyapatite substitute: a large-animal study." Tissue engineering. Part A vol. 21,3-4 (2015): 704-15
Biomat + cells	Monolayer	/	Fibrin	/	/	Other MSCs (menstrual MSCs)	/	/	Malleable	Rabbit	X	X	X			Khanmohammadi, Manijeh et al. "Repair of Osteochondral Defects in Rabbit Knee Using Menstrual Blood Stem Cells Encapsulated in Fibrin Glue: A Good Stem Cell Candidate for the Treatment of Osteochondral Defects." Tissue engineering and regenerative medicine vol. 16,3 311-324. 27 Apr. 2019,
Biomat + cells	Monolayer	/	Gelatin + alginate	/	/	Articular chondrocytes	/	/	Other	Rabbit	X		X	X		Nie, Xiaolei et al. "Full-Scale Osteochondral Regeneration by Sole Graft of Tissue-Engineered Hyaline Cartilage without Co-Engraftment of Subchondral Bone Substitute." Advanced healthcare materials vol. 9,2 (2020): e1901304.
Biomat + cells	Bilayer	Biomat + cells	Alginate + CS-AEMA + GelMA	Alginate + CS-AEMA + GelMA + HA-MA	/	BM-MSCs + articular chondrocytes	/	Press-fit	3D-printing	Rat	X	X	X			Idaszek, Joanna et al. "3D bioprinting of hydrogel constructs with cell and material gradients for the regeneration of full-thickness chondral defect using a microfluidic printing head." Biofabrication vol. 11,4 044101. 1 Jul. 2019,

Biomat + cells	Bilayer	Biomat + cells	HA + poly-glycidol + PCL	/	PCL	BM-MSCs + articular cartilage progenitor cells (ACPC)	/	Press-fit	3D-printing	Poney	X	X	X	X	X	Mancini, I A D et al. "A composite hydrogel-3D printed thermoplast osteochondral anchor as example for a zonal approach to cartilage repair: in vivo performance in a long-term equine model." Biofabrication vol. 12,3 035028. 1 Jul. 2020,
Biomat + cells	Bilayer	Biomat + cells	Agarose + alginate	/	Alginate + BG	BM-MSCs (bone) BM-MSCs + articular chondrocytes (cartilage)	/	/	Injection	Rat	X	X	X	X		Zhu, Yanlun et al. "An injectable continuous stratified structurally and functionally biomimetic construct for enhancing osteochondral regeneration." Biomaterials vol. 192 (2019): 149-158.
Biomat + cells	Monolayer	/	PEG vinyl sulfone	/	/	Chondrocytes	/	/	Injection	Mouse	X					Wang, Jianqi et al. "Fabrication of injectable high strength hydrogel based on 4-arm star PEG for cartilage tissue engineering." Biomaterials vol. 120 (2017): 11-21
Biomat + cells	Monolayer	/	PLLA + Gelatin	/	/	BM-MSCs (differentiated)	/	/	Electrospinning	Rabbit	X			X		Jin, Le et al. "Osteochondral tissue regenerated via a strategy by stacking pre-differentiated BMSC sheet on fibrous mesh in a gradient." Biomedical materials (Bristol, England) vol. 14,6 065017. 8 Nov. 2019,
Biomat + cells	Monolayer	/	Col I	/	/	Mixed cells (from bone marrow)	/	/	Commercial	Rabbit	X	X				Veronesi, Francesca et al. "Pulsed electromagnetic fields combined with a collagenous scaffold and bone marrow concentrate enhance osteochondral regeneration: an in vivo study." BMC musculoskeletal disorders vol. 16 233. 2 Sep. 2015,
Biomat + cells	Monolayer	/	HA	/	/	UCB-MSCs	/	/	Injection	Minipig	X	X	X			Wu, Kun-Chi et al. "Transplanting human umbilical cord mesenchymal stem cells and hyaluronate hydrogel repairs cartilage of osteoarthritis in the minipig model." Ci ji yi xue za zhi = Tzu-chi medical journal vol. 31,1 (2019): 11-19.
Biomat + cells	Monolayer	/	PLGA	/	/	BM-MSCs or Adip-MSCs or chondrocyte progenitor cells	/	Press-fit	Molding	Sheep	X	X	X			Caminal, M et al. "Cartilage resurfacing potential of PLGA scaffolds loaded with autologous cells from cartilage, fat, and bone marrow in an ovine model of osteochondral focal defect." Cytotechnology vol. 68,4 (2016): 907-19
Biomat + cells	Monolayer	/	Fibrin	/	/	Embryonic stem-like cells (ESL)	/	/	Malleable	Sheep	X	X	X		X	Manunta, Andrea Fabio et al. "The use of embryonic cells in the treatment of osteochondral defects of the knee: an ovine in vivo study." Joints vol. 4,2 70-9. 18 Aug. 2016,
Biomat + cells	Monolayer	/	Dextran	/	/	Adip-MSCs or chondrocytes	/	/	Injection	Rabbit	X	X	X			Fan, Lin et al. "An Injectable Bioorthogonal Dextran Hydrogel for Enhanced Chondrogenesis of Primary Stem Cells." Tissue engineering. Part C, Methods vol. 24,9 (2018): 504-513.
Biomat + cells	Monolayer	/	Col I	/	/	Nasal chondrocytes	/	/	Injection	Rat	X	X	X			Lim, Mi Hyun et al. "Evaluation of Collagen Gel-Associated Human Nasal Septum-Derived Chondrocytes As a Clinically Applicable Injectable Therapeutic Agent for Cartilage Repair." Tissue engineering and regenerative medicine vol. 17,3 (2020): 387-399.
Biomat + cells	Monolayer	Biomat + cells	Cartilage ECM + Col	/	/	Articular chondrocytes	/	/	Other	Pork	X		X			Meppelink, Amanda M et al. "Hyaline Articular Matrix Formed by Dynamic Self-Regenerating Cartilage and Hydrogels." Tissue engineering. Part A vol. 22,13-14 (2016): 962-70.
Biomat + cells	Bilayer	Biomat + cells	Agarose or alginate	/	Alginate + PCL or agarose	BM-MSCs (bone) Chondrocytes + Adip-MSCs (infrapatellar) (cartilage)	/	/	3D-printing + Molding	Goat	X		X			Critchley, Susan et al. "3D printing of fibre-reinforced cartilaginous templates for the regeneration of osteochondral defects." Acta biomaterialia vol. 113 (2020): 130-143.
Biomat + cells	Monolayer	/	Col I	/	/	Nasal chondrocytes	/	/	Injection	Rat	X		X			Lim, Mi Hyun et al. "Evaluation of Collagen Gel-Associated Human Nasal Septum-Derived Chondrocytes As a Clinically Applicable Injectable Therapeutic Agent for Cartilage Repair." Tissue engineering and regenerative medicine vol. 17,3 (2020): 387-399.
Biomat + cells	Monolayer	/	Col I + Col II	/	/	BM-MSCs	/	Press-fit	Undefined	Rabbit	X	X				Kilmer, Claire E et al. "Collagen Type I and II Blend Hydrogel with Autologous Mesenchymal Stem Cells as a Scaffold for Articular Cartilage Defect Repair." ACS biomaterials science & engineering vol. 6,6 (2020): 3464-3476.
Biomat + cells	Monolayer	/	Cartilage ECM	/	/	Adip-MSCs	/	Fibrin glue	Molding	Rabbit	X	X				Widhiyanto, Lukas et al. "Macroscopic and histologic evaluation of cartilage regeneration treated using xenogenic biodegradable porous sponge cartilage scaffold composite supplemented with allogenic adipose derived mesenchymal stem cells (ASCs) and secretome: An in vivo experimental study." Journal of biomaterials applications vol. 35,3 (2020): 422-429.
Biomat + cells	Monolayer	/	Cartilage ECM + gelatin	/	/	Adip-MSCs	/	/	Punch	Rabbit	X			X		Tsai, Ching-Cheng et al. "Enzyme-Cross-linked Gelatin Hydrogel Enriched with an Articular Cartilage Extracellular Matrix and Human Adipose-Derived Stem Cells for Hyaline Cartilage Regeneration of Rabbits." ACS biomaterials science & engineering vol. 6,9 (2020): 5110-5119.
Biomat + cells	Trilayer	Biomat	Chitosan + gelatin	Chitosan + gelatin	/	BM-MSCs	/	Press-fit	Molding	Rabbit	X	X	X			Rajagopal, Karthikeyan et al. "In vivo cartilage regeneration in a multi-layered articular cartilage architecture mimicking scaffold." Bone & joint research vol. 9,9 601-612. 23 Sep. 2020
Biomat + cells	Monolayer	/	PLGA + TCP	/	/	Adip-MSCs (infrapatellar fat pad)	/	/	Undefined	Rabbit	X	X	X			Lu, Zhihua et al. "Unfavorable Contribution of a Tissue-Engineering Cartilage Graft to Osteochondral Defect Repair in Young Rabbits." Frontiers in cell and developmental biology vol. 8 595518. 29 Oct. 2020,
Biomat + cells	Trilayer	Biomat	Silk fibroin	Silk fibroin + HAP	Silk fibroin + HAP	Adip-MSCs	/	/	Molding	Rabbit	X	X	X		X	Zhao, Yanhong et al. "Role of the Calcified Cartilage Layer of an Integrated Trilayered Silk Fibroin Scaffold Used to Regenerate Osteochondral Defects in Rabbit Knees." ACS biomaterials science & engineering vol. 6,2 (2020): 1208-1216
Biomat + cells	Monolayer	/	PCL	/	/	Other MSCs (foetal MSCs)	/	Fibrin glue	Other	Rabbit	X	X	X			Lin, Y. M.et al. "Human early mesenchymal stromal cells delivered on porous lightweight biodegradable polycaprolactone-based microcarriers result in improved cartilage formation." Materialia, (2020) 13, 100851.
Biomat + cells	Bilayer	Biomat	PCL + PVA	/	BG	Chondrocytes	/	Fibrin glue	Undefined	Rabbit	X	X				Rajagopal, Karthikeyan et al. "Long-Term Evaluation of Allogenic Chondrocyte-Loaded PVA-PCL IPN Scaffolds for Articular Cartilage Repair in Rabbits." Indian journal of orthopaedics vol. 55,4 853-860. 3 Jan. 2021,
Biomat + cells	Monolayer	/	Cartilage ECM + PCL + PGMA	/	/	Adip-MSCs	/	/	Malleable	Rat	X	X	X	X		Kim, Hye Sung et al. "Protein-reactive nanofibrils decorated with cartilage-derived decellularized extracellular matrix for osteochondral defects." *Biomaterials* vol. 269 (2021): 120214.
Biomat + cells	Monolayer	/	Silk firboin	/	/	Articular chondrocytes	/	/	Injection	Rabbit	X	X	X		X	Yuan, Tao et al. "Injectable Ultrasonication-Induced Silk Fibroin Hydrogel for Cartilage Repair and Regeneration." *Tissue engineering. Part A* vol. 27,17-18 (2021): 1213-1224.
Biomat + cells	Bilayer	Biomat + cells	Col I + HA	Col I + HA ??	BCP	Articular chondrocytes + BM-MSCs	/	/	Molding	Rabbit	X	X	X	X		Wang, Hongzhe et al. "Cell-mediated injectable blend hydrogel-BCP ceramic scaffold for in situ condylar osteochondral repair." Acta biomaterialia vol. 123 (2021): 364-378.
Biomat + cells	Bilayer	Biomat + cells	/	/	Bone ECM	Chondrocytes	/	/	?	?	X		X			Wongin, Sopita et al. "Evaluation of osteochondral-like tissues using human freeze-dried cancellous bone and chondrocyte sheets to treat osteochondral defects in rabbits." Biomaterials science vol. 9,13 (2021): 4701-4716
Biomat + cells	Trilayer	Biomat + cells	Col	Calcified cartilage ECM	Subchondral bone ECM	BM-MSCs	/	/	Other	Minipig	X	X	X			Huang, Yang et al. "Scaffold With Natural Calcified Cartilage Zone for Osteochondral Defect Repair in Minipigs." The American journal of sports medicine vol. 49,7 (2021): 1883-1891.
Biomat + cells	Monolayer	/	Fibrin	/	/	Articular chondrocytes	/	/	Malleable	Rabbit	X	X	X	X	X	Yang, Yu et al. "Porcine fibrin sealant combined with autologous chondrocytes successfully promotes full-thickness cartilage regeneration in a rabbit model." Journal of tissue engineering and regenerative medicine vol. 15,9 (2021): 776-787.
Biomat + cells	Bilayer	Biomat	PCL	/	PCL + TCP	Adip-MSCs	/	Press-fit	3D-printing + electrospinning	Minipig	X	X	X	X		Nordberg, Rachel C et al. "The evaluation of a multiphasic 3D-bioplotted scaffold seeded with adipose derived stem cells to repair osteochondral defects in a porcine model." Journal of biomedical materials research. Part B, Applied biomaterials vol. 109,12 (2021): 2246-2258
Biomat + cells	Bilayer	Biomat	Col I + Col III	Col I + Col III	/	Nasal chondrocyes	/	Press-fit	Commercial	Sheep	X	X	X			Šećerović, Amra et al. "Nasal Chondrocyte-Based Engineered Grafts for the Repair of Articular Cartilage "Kissing" Lesions: A Pilot Large-Animal Study." The American journal of sports medicine vol. 49,8 (2021): 2187-2198.
Biomat + cells	Monolayer	/	Col I + HAP + PVA	/	/	Chondrocytes	/	/	Molding	Goat	X		X			Xie, Jie et al. "Fabrication and characterization of microstructure-controllable COL-HA-PVA hydrogels for cartilage repair." Journal of materials science. Materials in medicine vol. 32,9 100. 18 Aug. 2021,
Biomat + cells	Bilayer	Biomat	Cellulose + chitosan	Chitosan + HAP	/	Other MSCs (buccal fat pad)	/	/	Molding	Rabbit	X		X			Dehghani Nazhvani, Fatemeh et al. "Effects of in vitro low oxygen tension preconditioning of buccal fat pad stem cells on in Vivo articular cartilage tissue repair." Life sciences vol. 280 (2021): 119728.
Biomat + cells	Monolayer	/	Chitosan + HA	/	/	Adip-MSCs	Chondrocyte extravesicles	/	?	Rabbit	X					Heirani-Tabasi, Asieh et al. "Cartilage tissue engineering by co-transplantation of chondrocyte extracellular vesicles and mesenchymal stem cells, entrapped in chitosan-hyaluronic acid hydrogel." Biomedical materials (Bristol, England) vol. 16,5 10.1088/1748-605X/ac0cbf. 13 Jul. 2021
Biomat + cells + biologics	Bilayer	Cells + biologics	Silk fibroin + GelMA	/	Silk fibroin-MA + GelMA	Articular chondrocytes (cartilage) / BM-MSCs (bone)	PTH	/	3D-printing	Rabbit	X	X	X	X		Deng, Changxu et al. "3D bio-printed biphasic scaffolds with dual modification of silk fibroin for the integrated repair of osteochondral defects." Biomaterials science vol. 9,14 (2021): 4891-4903.
Biomat + cells + biologics	Monolayer	/	HA + Col I	/	/	BM-MSCs	Icariin	/	Molding	Rabbit	X		X			Yang, Jirong et al. "Icariin conjugated hyaluronic acid/collagen hydrogel for osteochondral interface restoration." Acta biomaterialia vol. 74 (2018): 156-167.
Biomat + cells + biologics	Bilayer	Biologics	HA-MA + GelMA	/	HA-MA + GelMA	BM-MSCs	Kartogenin, melatonin	/	Injection	Rabbit	X	X	X	X		Liu, Xuemin et al. "Molecular recognition-directed site-specific release of stem cell differentiation inducers for enhanced joint repair." Biomaterials vol. 232 (2020): 119644
Biomat + cells + biologics	Monolayer	/	GelMA	/	/	Other MSCs	Kartogenin, TGF-b1	Press-fit	Injection	Rat	X	X	X			Xu, Jianbin et al. "Injectable stem cell-laden supramolecular hydrogels enhance in situ osteochondral regeneration via the sustained co-delivery of hydrophilic and hydrophobic chondrogenic molecules." Biomaterials vol. 210 (2019): 51-61.
Biomat + cells + biologics	Monolayer	/	PLGA	/	/	Syn-MSCs	BMP-7	/	Electrospinning	Rabbit	X		X			Kim, Hee Jung et al. "Intra-articular delivery of synovium-resident mesenchymal stem cells via BMP-7-loaded fibrous PLGA scaffolds for cartilage repair." Journal of controlled release : official journal of the Controlled Release Society vol. 302 (2019): 169-180.
Biomat + cells + biologics	Bilayer	Biomat + cells + biologics	Alginate + HA	/	PCL	BM-MSCs	BMP-2	/	Electrospinning + injection	Rat, sheep	X	X		X		Keller, Laetitia et al. "Preclinical safety study of a combined therapeutic bone wound dressing for osteoarticular regeneration." Nature communications vol. 10,1 2156. 14 May. 2019,
Biomat + cells + biologics	Bilayer	Biomat + biologics	PLGA	/	PLGA	BM-MSCs	BMP-2	/	Electrospinning + Molding	Rabbit	X		X			Vayas, Raquel et al. "Evaluation of the effectiveness of a bMSC and BMP-2 polymeric trilayer system in cartilage repair." Biomedical materials (Bristol, England) vol. 12,4 045001. 4 Jul. 2017,

Biomat + cells + biologics	Monolayer	/	Laminin	/	/	BM-MSCs	IGF-1, TGF-b1	/	Malleable	Rabbit	X	X				Gugjoo, Mudasir Bashir et al. “Mesenchymal stem cells with IGF-1 and TGF- β1 in laminin gel for osteochondral defects in rabbits.” Biomedicine & pharmacotherapy = Biomedecine & pharmacotherapie vol. 93 (2017): 1165-1174.
Biomat + cells + biologics	Monolayer	/	PRF	/	/	BM-MSCs	PRF	Press-fit	Other	Dog	X	X				Kazemi, D et al. “Canine articular cartilage regeneration using mesenchymal stem cells seeded on platelet rich fibrin: Macroscopic and histological assessments.” Bone & joint research vol. 6,2 (2017): 98-107.
Biomat + cells + biologics	Bilayer	Biomat + cells	/	/	Porous tantalum	Other cells (periostal)	TGF-b1	Press-fit	Commercial	Sheep	X	X				Mrosek, E H et al. “Porous tantalum biocomposites for osteochondral defect repair: A follow-up study in a sheep model.” Bone & joint research vol. 5,9 (2016): 403-11.
Biomat + cells + biologics	Monolayer	/	Alginate	/	/	Adip-MSCs	PRP	Periostal flap	Malleable	Rabbit	X	X				Beigi, Mohammad-Hossein et al. “Activated platelet-rich plasma improves cartilage regeneration using adipose stem cells encapsulated in a 3D alginate scaffold.” Journal of tissue engineering and regenerative medicine vol. 12,6 (2018): 1327-1338.
Biomat + cells + biologics	Monolayer	/	Col I	/	/	BM-MSCs	IGF-1, TGF-b1	/	Undefined	Rabbit	X	X				Gugjoo, Mudasir Bashir et al. “Allogeneic mesenchymal stem cells and growth factors in gel scaffold repair osteochondral defect in rabbit.” Regenerative medicine vol. 15,2 (2020): 1261-1275.
Biomat + cells + biologics	Monolayer	/	Gelatin + b-TCP	/	/	BM-MSCs	BMP-2, PRP	Synovial flap + fibrin glue	Cut	Horse	X	X	X	X		Seo, Jong-Pil et al. “Effects of a synovial flap and gelatin/β-tricalcium phosphate sponges loaded with mesenchymal stem cells, bone morphogenetic protein-2, and platelet rich plasma on equine osteochondral defects.” Research in veterinary science vol. 101 (2015): 140-3
Biomat + cells + biologics	Bilayer	Biomat	PLCL	/	b-TCP + PLA	BM-MSCs	IL-8	Press-fit	Molding	Dog	X	X	X			Yoon, Dong Suk et al. “Synergistic Action of IL-8 and Bone Marrow Concentrate on Cartilage Regeneration Through Upregulation of Chondrogenic Transcription Factors.” Tissue engineering. Part A vol. 22,3-4 (2016): 363-74.
Biomat + cells + biologics	Monolayer	/	PEO + PU	/		BM-MSCs	SDF-1, Y27632 drug	/	3D-printing	Rabbit	X	X				Wen, Yi-Ting et al. “Biodegradable water-based polyurethane scaffolds with a sequential release function for cell-free cartilage tissue engineering.” Acta biomaterialia vol. 88 (2019): 301-313.
Biomat + cells + biologics	Bilayer	Biomat + biologics	HA + PCL	/	Col I + PCL	Other MSCs (turbinate-derived)	BMP-2, TGF-b	Press-fit	3D-printing	Rabbit	X	X	X			Shim, Jin-Hyung et al. “Three-dimensional bioprinting of multilayered constructs containing human mesenchymal stromal cells for osteochondral tissue regeneration in the rabbit knee joint.” Biofabrication vol. 8,1 014102. 4 Feb. 2016,
Biomat + cells + biologics	Bilayer	Biomat	Col I + PLA-PCL	/	TCP	BM-MSCs (transfected for COMP expression)	COMP	/	Electrospinning + punch + commercial	Rabbit	X	X	X	X	X	Wang, Chongyang et al. “Cartilage oligomeric matrix protein improves in vivo cartilage regeneration and compression modulus by enhancing matrix assembly and synthesis.” Colloids and surfaces. B, Biointerfaces vol. 159 (2017): 518-526.
Biomat + cells + biologics	Monolayer	/	Fibrin+ fibrinogen	/	/	BM-MSCs	Plasmid	/	Molding	Rabbit	X	X		X		Feng, Xue et al. “Age-Related Regeneration of Osteochondral and Tibial Defects by a Fibrin-Based Construct in vivo.” Frontiers in bioengineering and biotechnology vol. 8 404. 5 May. 2020,
Biomat + cells + biologics	Monolayer	Biomat	Col I + cartilage ECM	/	/	BM-MSCs	BMP-7	/	Cut	Dog	X	X	X	X		Sun, Jiachen et al. “A biphasic, demineralized, and Decellularized allograft bone-hydrogel scaffold with a cell-based BMP-7 delivery system for osteochondral defect regeneration.” Journal of biomedical materials research. Part A vol. 108,9 (2020): 1909-1921.
Biomat + cells + biologics	Bilayer	Biomat + biologics	Chitosan + PLGA +	/	Chitosan + HAP + PLGA	Adip-MSCs	BMP-2	/	Undefined	Rabbit	X	X	X		X	Zhang, Kunxi et al. “Regeneration of hyaline-like cartilage and subchondral bone simultaneously by poly(l-glutamic acid) based osteochondral scaffolds with induced autologous adipose derived stem cells.” Journal of materials chemistry. B vol. 4,15 (2016): 2628-2645.
Biomat + cells + biologics	Monolayer	/	HA	/	/	Adip-MSCs (infrapatellar fat pad)	Matrilin-3	/	Malleable	Rat	X	X				Muttigi, Manjunatha S et al. “Matrilin-3 codelivery with adipose-derived mesenchymal stem cells promotes articular cartilage regeneration in a rat osteochondral defect model.” Journal of tissue engineering and regenerative medicine vol. 12,3 (2018): 667-675.
Biomat + cells + biologics	Monolayer	/	Alginate	/	/	Adip-MSCs	BMP-4	/	Injection	Pork	X	X	X	X	X	Chen, Linxin et al. “CaAlg hydrogel containing bone morphogenetic protein 4-enhanced adipose-derived stem cells combined with osteochondral mosaicplasty facilitated the repair of large osteochondral defects.” Knee surgery, sports traumatology, arthroscopy : official journal of the ESSKA vol. 27,11 (2019): 3668-3678.
Biomat + cells + biologics	Trilayer	Biomat + biologics	Cartilage ECM + silk fibroin	nHAP + silk fibroin	nHAP + silk fibroin	UCB-MSCs	BMP-2, TGF-b3	/	Molding	Rabbit	X	X	X	X		Dong, Yunsheng et al. "Regional and sustained dual-release of growth factors from biomimetic tri-layered scaffolds for the repair of large-scale osteochondral defects." Applied Materials Today, 19, (2020) 100548.
Biomat + cells + biologics	Bilayer	Biomat + cells + biologics	Alginate + HA	/	Col	BM-MSCs	BMP-2	/	Punch + Malleable	Sheep	X	X		X		Favreau, Henri et al. “Osteochondral repair combining therapeutics implant with mesenchymal stem cells spheroids.” Nanomedicine : nanotechnology, biology, and medicine vol. 29 (2020): 102253.
Biomat + cells + biologics	Monolayer	/	Placental MEC	/	/	BM-MSCs	PRP	/	Cut	Rat	X					Özdemir, Erdi et al. “Articular Cartilage Regeneration Utilizing Decellularized Human Placental Scaffold, Mesenchymal Stem Cells and Platelet Rich Plasma.” Tissue engineering and regenerative medicine vol. 17,6 (2020): 901-908
Biomat + cells + biologics	Monolayer	/	AH-MA + PLGA + Poly-L-lysine	/	/	Adip-MSCs	Kartogenin	/	Undefined	Rabbit	X	X				Hong, Yuping et al. “Combination Therapy Using Kartogenin-Based Chondrogenesis and Complex Polymer Scaffold for Cartilage Defect Regeneration.” ACS biomaterials science & engineering vol. 6,11 (2020): 6276-6284.
Biomat + cells + biologics	Monolayer	/	Gelatine-SH + PEGDA	/	/	Adip-MSCs	IGF-1	/	Molding	Rabbit	X	X				Cho, Hyeran et al. “Dual delivery of stem cells and insulin-like growth factor-1 in coacervate-embedded composite hydrogels for enhanced cartilage regeneration in osteochondral defects.” Journal of controlled release : official journal of the Controlled Release Society vol. 327 (2020): 284-295.
Biomat + cells + biologics	Monolayer	/	Col + HA + fibrinogen	/	/	Syn-MSCs	Transglutaminase-4	/	Injection	Rabbit	X	X	X			Kim, Jong-Keun et al. “Enhancement of Cartilage Regeneration of Synovial Stem Cells/Hydrogel by Using Transglutaminase-4.” Tissue engineering. Part A vol. 27,11-12 (2021): 761-770.
Biomat + cells + biologics	Trilayer	Biomat	GelMA + PCL + PEG	GelMA + PCL + PEG	GelMA + PCL + PEG	BM-MSCs	BMP-2, BMP-7, TGF-b1	/	Molding + 3D-printing	Rabbit	X	X	X	X	X	Qiao, Zhiguang et al. “Bioinspired stratified electrowritten fiber-reinforced hydrogel constructs with layer-specific induction capacity for functional osteochondral regeneration.” Biomaterials vol. 266 (2021): 120385.
Biomat + cells + biologics	Monolayer	/	Col	/	/	BM-MSCs	HIF-1a		?	Rabbit	X	X	X	X		Cheng, Mo-Sha et al. “Overexpression of HIF-1alpha in Bone Marrow Mesenchymal Stem Cells Promote the Repair of Mandibular Condylar Osteochondral Defect in a Rabbit Model.” Journal of oral and maxillofacial surgery : official journal of the American Association of Oral and Maxillofacial Surgeons vol. 79,2 (2021): 345.e1-345.e15.
Biomat + cells + biologics	Trilayer	Biomat	Col or PLGA	Col + HAP or PLA	Col + HAP or titanium alloy	BM-MSCs	Bone marrow aspirate concentrate	Press-fit	Molding (scaffold 1) 3D-printing + Molding (scaffold 2)	Sheep		X		X		Flaherty, Taylor et al. "Micro-Computed Tomography Analysis of Subchondral Bone Regeneration Using Osteochondral Scaffolds in an Ovine Condyle Model". Appl. Sci. 2021, 11, 891
Biomat + cells + biologics	Monolayer	/	Alginate	/	/	BM-MSCs	Bone marrow aspirate concentrate	/	Malleable	Rabbit	X	X	X	X	X	Xu, Liang et al. “Ultrapurified Alginate Gel Containing Bone Marrow Aspirate Concentrate Enhances Cartilage and Bone Regeneration on Osteochondral Defects in a Rabbit Model.” The American journal of sports medicine vol. 49,8 (2021): 2199-2210.
Biomat + cells + biologics	Monolayer	/	Alginate	/	/	BM-MSCs	Ghrelin, TGF-b3	/	Undefined	Rat	X	X	X			Li, Kejia et al. “Nanosecond pulsed electric fields prime mesenchymal stem cells to peptide ghrelin and enhance chondrogenesis and osteochondral defect repair in vivo.” Science China. Life sciences, 10.1007/s11427-021-1983-y. 23 Sep. 2021,
Biomat + graft	Monolayer	/	Alginate	/	/	Mixed cells	Mixed biologics	/	Undefined	Rabbit	X	X	X	X		Hishimura, Ryosuke et al. “Osteochondral Autograft Transplantation Technique Augmented by an Ultrapurified Alginate Gel Enhances Osteochondral Repair in a Rabbit Model.” The American journal of sports medicine vol. 47,2 (2019): 468-478
Biomat + graft	Trilayer	Biomat + cells	PLGA	PLGA	Titanium alloy	Articular chondrocytes (titane implant) or articular chondrocytes (cartilage) and BM-MSCs (bone)	BMP-2; TGF-b3	Press-fit	3D-printing + bioreactor	Goat	X	X	X		X	Zhai, Chenjun, et al. "Utilizing an integrated tri-layered scaffold with Titanium-Mesh-Cage base to repair cartilage defects of knee in goat model." Materials & Design, 193, (2020) 108766
Cultured graft	Bilayer	Biomat	Col I	/	Col I + HAP	Nasal chondrocytes	Mixed biologics (cultured graft)	Press-fit	Other	Sheep	X	X	X			Vukasovic, Andreja et al. “Bioreactor-manufactured cartilage grafts repair acute and chronic osteochondral defects in large animal studies.” *Cell proliferation* vol. 52,6 (2019): e12653.
Cultured graft	Trilayer	Biomat	Agarose	Agarose + bone ECM	Bone ECM	Articular chondrocytes	Mixed biologics	Press-fit	Molding	Dog	X	X	X			Stefani, Robert M et al. “Pulsed electromagnetic fields promote repair of focal articular cartilage defects with engineered osteochondral constructs.” *Biotechnology and bioengineering* vol. 117,5 (2020): 1584-1596.
Cultured graft	Monolayer	/	PLA + PGA	/	/	BM-MSCs	/	Suture	Molding	Minipig	X	X	X		X	He, Aijuan et al. “Repair of osteochondral defects with in vitro engineered cartilage based on autologous bone marrow stromal cells in a swine model.” *Scientific reports* vol. 7 40489. 13 Jan. 2017,

Abbreviations

Adip-MSCs	Adipose-derived mesenchymal stromal cells
Amniotic MSCs	Amniotic mesenchymal stromal cells
b-TCP	Beta-tricalcium phosphate
BG	Bioactive glass
Biomat	Biomaterial
BM-MSCs	Bone marrow-derived mesenchymal stromal cells
BMP-2	Bone morphogenic protein-2
BMP-4	Bone morphogenic protein-4

BMP-7	Bone morphogenic protein-7
CaP	Calcium Phosphate
Col	Collagen
Col I	Collagen type I
Col II	Collagen type II
Col III	Collagen type III
Col X	Collagen type X
COMP	Cartilage oligomeric protein
CS	Chondroitin sulfate
CS-AEMA	Chondroitin sulfate-aminoethyl methacrylate
ECM	Extracellular matrix
FGF-2	Fibroblast growth factor-2
GAG	Glycosaminoglycan
GelMA	Gelatin-methacrylate
Glycidyl-MA	Glycidyl-methacrylate
HA	Hyaluronic acid
HA-MA	Hyaluronic acid-methacrylate
HAP	Hydroxyapatite
Histo	Histology
IGF-1	Insulin growth factor-1
IHC	Immunohistochemistry
IL-4	Interleukin-4
IL-8	Interleukin-8
Macro. Obs.	Macroscopic observation
Micro-CT	Micro-computed tomography
MIP-3a	Macrophage inflammatory protein-3 alpha
MSCs	Mesenchymal stromal cells
OPF	Oligo(polyethylene glycol) fumarate
PA6	Polyamide 6
PACG	Poly(N-acryloyl 2-glycine)
PAMPS	Poly-(2-acrylamido-2-methylpropanesulfonic acid)
PBT	Poly(butylene terephtalate)
PCL	Poly(caprolactone)
PDGF	Platelet-derived growth factor
PDMAAm	Poly-(N,N'-dimethyl acrylamide)
PEEK	Polyetheretherketone
PEG-MA	Poly(ethylene glycol)-methacrylate
PEGDA	Poly(ethylene glycol)-diacrylate
PEO	Poly(ethylene oxide)
PEOT	Poly(ethyleneoxide)-terephtalate
PGA	Poly(glycolic acid)
PLA	Poly(lactic acid)
PLCL	Poly (l-lactide-co-ε-caprolactone)
PLGA	Poly(lactic-co-glycolic acid)
PNAGA	Poly(N-acryloyl glycinamide)
PNIPAAm	poly(N-isopropylacrylamide)
PRF	Platelet-rich fibrin
PRP	Platelet-rich plasma
PSPU-U	Polyester polyurethane–urea
PTB	Polybutylene-terephthalate
PTH	Parathyroid hormone
PU	Polyurethane
PVA	Poly vinyl alcohol
rAAV	Recombinant adeno associated virus
SDF-1	Stromal-derived factor-1
Syn-MSCs	Synovial mesenchymal stromal cells
TCP	Tricalcium phosphate
TGF-b1	Transforming growth factor beta1
TGF-b3	Transforming growth factor beta3
UCB-MSCs	Umbilical cord blood-derived mesenchymal stromal cells
Wnt5a	Wingless type 5a
